# Ginsenoside Rg3-encapsulated pegylated niosomes exhibit multimodal therapeutic potential in Alzheimer’s disease

**DOI:** 10.1038/s41598-025-32528-3

**Published:** 2025-12-14

**Authors:** Mahmood Barani, Farshid Zargari, Shekoufeh Mirinejad, Fatemeh Madani, Mohammad Reza Hajinezhad, Saman Sargazi

**Affiliations:** 1 Department of Chemistry, Faculty of Nano and Bio Science and Technology, Persian Gulf University, Bushehr, 75168 Iran; 2 Department of Chemistry, Faculty of Science, University of Sistan and Balouchestan, Zahedan, Iran; 3 Cellular and Molecular Research Center, Research Institute of Cellular and Molecular Sciences in Infectious Diseases, Zahedan University of Medical Sciences, Zahedan, Iran; 4 Department of Medical Nanotechnology, School of Advanced Technologies in Medicine, Tehran University of Medical Sciences, Tehran, Iran; 5 Basic Science Department, Faculty of Veterinary Medicine, University of Zabol, Zabol, Iran; 6 Genetics of Non-Communicable Disease Research Center, Zahedan University of Medical Sciences, Zahedan, Iran; 7 Department of Clinical Biochemistry, School of Medicine, Zahedan University of Medical Sciences, Zahedan, Iran

**Keywords:** Niosome, Ginsenoside Rg3, Alzheimer’s disease, Drug delivery, Nanoparticle, Molecular dynamics, Biochemistry, Drug discovery, Neuroscience

## Abstract

Ginsenoside Rg3 (GRg3), a bioactive compound extracted from ginseng, has demonstrated the ability to inhibit Aβ production and deposition. In this study, PEGylated GRg3-loaded niosomes were developed and characterized for potential AD treatment. Their efficacy was assessed using *in vitro* and *in vivo* models, as well as molecular dynamics simulations of self-assembly. Our formulation achieved a relatively high encapsulation efficiency of 83.02% and a controlled release profile, with 75.73% of the drug released over 48 h. *In vitro*, co-administration of Aβ with free or PEGylated GRg3-loaded niosomes markedly reduced the levels of Total Antioxidant Capacity, Malondialdehyde (MDA), and *caspase-3* gene expression compared to the Aβ-only group. *In vivo* evaluations revealed that treatment with the niosomal formulation did not significantly alter behavioral parameters, MDA levels, or Superoxide Dismutase activity. However, catalase activity was significantly higher than in the control group. Histopathological and immunohistochemical analyses showed reduced neurovascular damage and preservation of blood–brain barrier (BBB) and hippocampal integrity in the treated group. MD simulations confirmed the spontaneous self-assembly of surfactant molecules into a bilayer structure with successful incorporation of GRg3. Our findings underscore the potential of PEGylated niosomes as efficient nanocarriers for GRg3 delivery in the AD treatment.

**Supplementary Information:**

The online version contains supplementary material available at 10.1038/s41598-025-32528-3.

## Introduction

Alzheimer’s disease (AD) is considered one of the leading obstacles in the field of neurodegenerative conditions, affecting approximately 50 million individuals, primarily among the elderly population. With the continuous growth of aging demographics, this number is projected to increase substantially^1^. Clinically, AD presents with cognitive impairments, including memory loss, disorientation, and attention deficits, often necessitating continuous care from family members and professional caregivers^2,3^. Pathologically, AD is marked by neuroinflammation, extracellular amyloid beta (Aβ) plaque accumulation, and intracellular tau protein hyperphosphorylation^4,5^. Two principal theories explain the pathogenesis of AD: the cholinergic hypothesis, which implicates deficits in acetylcholine neurotransmission, and the Aβ hypothesis, which highlights the neurotoxic effects of Aβ aggregation^6–8^. Furthermore, several contributing risk factors have been identified, including aging, genetic susceptibility, traumatic brain injuries, cerebrovascular conditions, infections, and environmental exposures^9^.

Aβ plays a fundamental role in the pathogenesis of AD, contributing to neuronal dysfunction and progressive cognitive decline. One of the principal mechanisms by which Aβ induces neurotoxicity is through the overproduction of reactive oxygen species (ROS). This oxidative stress is primarily initiated by Aβ-induced mitochondrial dysfunction, where the efficiency of the electron transport chain is disrupted. As a result, excess electrons leak from the mitochondria and react with molecular oxygen to create ROS, such as hydroxyl radicals, superoxide anions, and hydrogen peroxide^10^. Simultaneously, Aβ impairs the function and expression of endogenous antioxidant defense systems, including enzymes such as catalase, superoxide dismutase (SOD), and glutathione peroxidase. The imbalance between ROS production and antioxidant capacity leads to a harmful oxidative environment within neurons^11^. Elevated ROS levels trigger a cascade of cellular damage, including lipid peroxidation (LPO), protein oxidation, and DNA damage. These processes compromise membrane integrity, impair neuronal signaling, and induce genomic instability, ultimately leading to synaptic dysfunction, neuroinflammation, and neuronal death; hallmarks of AD progression^12^.

Given the critical role of Aβ-induced oxidative stress in AD progression, antioxidants have emerged as promising therapeutic agents. While endogenous enzymes such as SOD, catalase, and glutathione help neutralize ROS^13,14^, their function is impaired in AD, thereby exacerbating oxidative damage. Thus, exogenous antioxidants, including herbal-based compounds, offer potential neuroprotective benefits by scavenging ROS, modulating Aβ aggregation, and reducing neuroinflammation^13^.

*Panax ginseng* C.A. Mey (P. ginseng), a renowned plant from the Araliaceae family, has been traditionally used in herbal medicine, demonstrating potential in antioxidant, anticancer, anti-inflammatory^16^, and memory-improving effects^14^. P. ginseng contains a variety of bioactive compounds, including phenolic compounds, ginsenosides, polyacetylenes, polysaccharides, alkaloids, peptides, fatty acids, amino acids, and vitamins^15^. Ginsenosides possess strong neuroprotective features associated with AD, such as inhibition of Aβ production and deposition, thereby mitigating the hallmark of AD pathology^16–18^. Among ginsenosides, ginsenoside Rg3 (GRg3) has garnered significant attention due to its neuroprotective effects^19^. Studies suggest that GRg3 is capable of mitigating oxidative stress through its scavenging potential of free radicals and activation of endogenous antioxidant enzymes. Furthermore, it is stated that GRg3 reduces Aβ aggregation, inhibits tau hyperphosphorylation, and improves synaptic plasticity, which are implicated in the commencement and progression of AD^20^. Despite its potential effectiveness, challenges such as meager solubility in water, poor bioavailability, and limited blood-brain barrier (BBB) penetration highlight the need for the development of an efficient drug delivery system^20^.

Recently, the application of nanoparticles (NPs) in drug delivery systems has revolutionized therapeutic strategies for treating AD. Among various types of NPs, niosomes are non-ionic surfactant vesicles that provide a promising framework for targeted and controlled drug release^21^. Niosomes are particularly advantageous due to their ability to encapsulate both hydrophilic and lipophilic drugs, thereby enhancing drug bioavailability and minimizing the side effects often associated with conventional delivery methods^22^. Compared to other lipid-based NPs, such as micelles and liposomes, niosomes offer greater stability, making them a more reliable platform for improving therapeutic efficacy. Additionally, niosomes can effectively cross the BBB, ensuring higher local drug concentrations within the central nervous system (CNS) while reducing systemic toxicity^23–25^. Polyethylene glycol (PEG) is a commonly used systemic polymer that modifies the surface of NPs, thereby significantly improving their pharmacokinetic properties. It is evident that PEG coating prevents the rapid clearance of NPs by the immune system, enhances the stability of the NPs, and improves their ability to cross the BBB^26^.

This research aimed to design PEGylated GRg3-loaded niosomes and evaluate their efficiency in treating an animal model of AD for the first time. GRg3 was encapsulated within PEGylated niosomes, and the physicochemical properties of the niosomes were assessed. Moreover, its neuroprotective effects were analyzed using cellular viability assays, MDA, TAC, and *caspase-3* expression analysis. In addition, the effectiveness of the PEGylated GRg3-loaded niosome was demonstrated in an AD model using Wistar rats. Finally, in silico analyses were performed to predict the interactions between GRg3 and the niosomal phospholipid bilayer, and to evaluate GRg3’s penetration efficiency, providing insights for optimizing its delivery through niosomal formulations for the treatment of AD.

## Material and method

### Chemicals and assay kits

GRg3 (purity ≥ 98%) (Catalog No. A14518) was procured from Adooq Bioscience (LLC, Irvine, CA, US), PEG (6000, MW: 5400 g/mol), Aβ 1–42 peptide, and 3-(4,5-dimethylthiazol-2-yl)−2,5-diphenyltetrazolium bromide (MTT) powder were purchased from Sigma-Aldrich (Germany). CHL (≥ 99% purity), Span 40, Tween 40, and chloroform (≥ 99.9% purity) were obtained from Merck (Germany). Fetal bovine serum (FBS), trypsin-EDTA solution (0.25%), and dimethyl sulfoxide (DMSO) were purchased from Gibco (Thermo Fisher Scientific, Germany). Penicillin-streptomycin solution (100 U/mL penicillin, 100 µg/mL streptomycin) and phosphate-buffered saline (PBS) tablets (pH 7.4) were obtained from Inoclon (Iran). The cDNA Synthesis Kit was obtained from ParsTous (Iran). SYBR Green Master Mix (High ROX) was purchased from Amplicon (Denmark). Amyloid β Protein Fragment 1–42 (Aβ1–42, Cat. No. A9810) was purchased from Sigma-Aldrich (Merck, Germany). The human neuroblastoma cell line SH-SY5Y was obtained from the Pasteur Institute of Iran’s Cell Bank. Male Wistar rats (220 ± 20 g) were procured from Zahedan University of Medical Sciences (Iran). Ketamine (10%) and xylazine (2%) were purchased from Iran Daru (Iran).

### Preparation of niosomes

PEGylated GRg3-loaded noisome were prepared using the thin-film hydration method, as described in previous studies with some modifications^27–29^. CHL, S40, and T40 were dissolved separately in chloroform, and then mixed under stirring at 200 rpm. GRg3 was dissolved in 1 mL of chloroform and gently mixed with the surfactants under stirring at 300 rpm at room temperature. The solution was then transferred into a 100 mL round-bottom flask, and the organic solvent was evaporated using a rotary evaporator (Heidolph, Germany) under reduced pressure at 60 °C with a water bath until a thin, dry lipid film was obtained. The film was then cooled to room temperature and hydrated in PBS (pH 7.4) for 30 min at 60 °C to yield a niosomal suspension. Once hydration was complete, the microtip sonicator was used for 45 min to further reduce the average vesicle size. Next, the PEG solution was added to the formulation, and the mixture was incubated at 37 °C for 24 h in a shaking incubator. To prepare niosomes without GRg3, the same procedure was followed, except for the addition of GRg3 (Figure S1). The free GRg3 was separated from the niosomes using a dialysis membrane (MW: 12,000, Sigma-Aldrich, USA) immersed in PBS for 1 h. The purified niosomes were then redispersed in deionized water and stored in an ultra-low freezer for 2 h before the freeze-drying process. The vacuum was set to 0.035 mbar, with the ice condenser set at − 81 °C and the shelf temperature at 10 °C. The resulting niosomal formulations were stored in a refrigerator for further analysis.

### Size and zeta measurement

Dynamic Light Scattering (DLS) measurements were performed using a Malvern Zetasizer to determine the hydrodynamic size and zeta potential of the synthesized NPs. Two different formulations were analyzed (PEGylated GRg3-loaded niosome and GRg3-loaded niosome). For each sample, the niosomes were dispersed in deionized water and sonicated for 10 min to ensure uniform dispersion. Measurements were conducted at room temperature (25 °C) with a scattering angle of 173° in a polystyrene cuvette. The hydrodynamic diameter (Z-average size) and zeta potential were recorded to assess the particle size distribution, while electrophoretic mobility measurements were used to determine the zeta potential.

### Morphology assessment

The morphological characteristics of both PEGylated GRg3-loaded niosome and GRg3-loaded niosome were analyzed using Transmission Electron Microscopy (TEM) (Philips, EM, 208 S). Samples were prepared by placing a drop of the niosome suspension on a carbon-coated copper grid, followed by negative staining and drying before imaging.

### Measurement of encapsulation efficiency (EE)

To determine the EE%, 1 mL of the synthesized PEGylated GRg3-loaded niosome was centrifuged at 15,000 rpm for 30 min at 4 °C to separate the free drug from the encapsulated drug. The supernatant containing unencapsulated GRg3 was carefully collected and analyzed using a UV-Vis spectrophotometer (Carry 60) at the absorption wavelength of GRg3. To determine the total GRg3 content, the niosomal formulation was disrupted using Triton X-100 solution and analyzed using the same spectrophotometric method. The EE% was then calculated using the formula:

### Drug release study

For the in vitro drug release study, a dialysis membrane was pre-soaked in PBS, pH 7.4, for at least 12 h before the experiment. 1 mL of PEGylated GRg3-loaded niosome was placed inside the dialysis bag, which was then appropriately sealed and immersed in 100 mL of PBS (pH 7.4) under continuous stirring at 100–150 rpm at 37 °C to mimic physiological conditions. At predetermined time intervals up to 48 h, 1 mL of the release medium was withdrawn and immediately replaced with an equal volume of fresh PBS to maintain sink conditions. The collected samples were analyzed using a UV-Vis spectrophotometer to determine the amount of drug released. The cumulative drug release (%) was calculated using the formula below. Finally, a drug release profile (percentage of drug released vs. time) was plotted to analyze the release kinetics, providing insight into the release behavior of PEGylated GRg3-loaded niosomes.

To understand the release mechanism, the release data were fitted to various kinetic models, including:


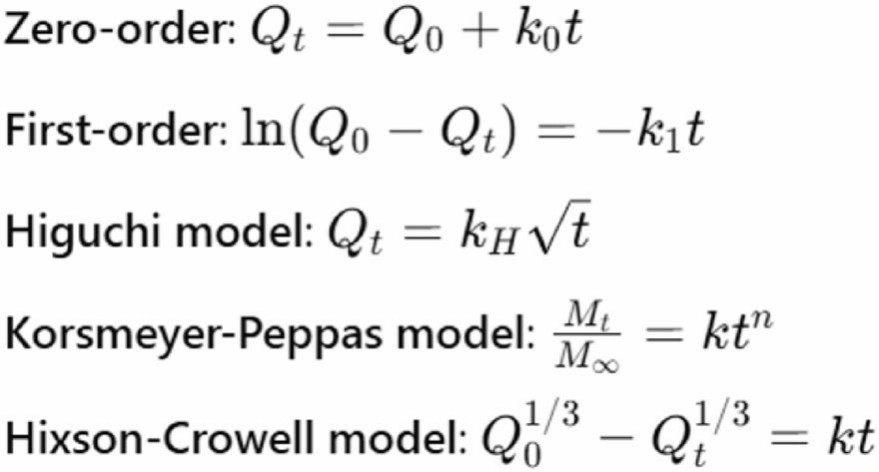


Here, Qt is the cumulative amount of drug released at time t, Q0​ is the initial drug load, Mt/M ∞ is the fraction of drug released, k is the rate constant, and n is the release exponent (in the Korsmeyer-Peppas model). Linear regression analysis was performed on the linearized forms of each model, and the best-fitting model was identified based on the coefficient of determination (R²).

### In vitro examinations

The SH-SY5Y cell cultures were maintained under standard culture conditions at 37 °C and 5% CO₂ before being proliferated in 96-well plates until they reached logarithmic growth. Cells received Aβ, free GRg3, and PEGylated GRg3-loaded niosomes through defined experimental conditions, as outlined in the methodological sections below, for in vitro assays.

#### Cytotoxicity evaluation

The SH-SY5Y cells received their specific treatment after achieving logarithmic growth in 96-well plates. The study utilized four experimental groups: (a) control group maintained for 24 h, (b) 10 µM Aβ exposure for 24 h, (c) 2.5 to 40 µM free GRg3 treatment, and (d) cells received 2.5 to 40 µM PEGylated GRg3-loaded niosome over 4 h before 24 h Aβ exposure (total incubation time of 28 h). After incubation, the wells received 20 µL of MTT solution at a concentration of 5 mg/mL and were maintained at 37 °C for an additional 4 h. The cell optical density was measured at a wavelength of 570 nm to determine cell survival rates using the previously mentioned formula^30^.

#### Malondialdehyde (MDA) assay

In the present study, the quantification of MDA, a derivative of LPO, was performed using a spectrophotometric assay based on its reaction with thiobarbituric acid, following the method described by Esterbauer and Cheeseman (1990)^31^. Intracellular MDA levels were assessed using a commercially purchased detection kit (Beyotime Biotechnology, S0131, Shanghai, China) according to the manufacturer’s instructions. Cells were randomized to one of two experimental treatment schedules: (1) continuous exposure to 10 µM Aβ, free GRg3, and/or PEGylated GRg3-loaded niosome for 28 h, or (2) pre-treatment for 4 h with free GRg3 or PEGylated niosomes (10 µM concentration), followed by co-treatment with 10 µM Aβ for an additional 24 h, for a total treatment duration of 28 h. Next, the cells were harvested, lysed, and centrifuged at 10,000 × g for 10 min to collect the supernatant. Then, standards and samples were prepared, and absorbance was read at 532 nm and 450 nm. MDA sample concentrations were calculated from a standard curve and expressed in nanomoles per milligram of protein.

#### Total antioxidant (TAC) assay

Following the treatment protocol established for MDA assay, we measured the total antioxidant capacity (TAC) using a TAC colorimetric assay kit (Zellbio GmbH, Germany). The assay kit measured the total reducing power of antioxidants between small molecules and proteins when they convert ferric ions (Fe³⁺) in the ferric-reducing ability of plasma (FRAP) solution to ferrous ions (Fe²⁺) under acidic conditions. The blue-colored complex formed by this reduction reaction enables absorbance measurement at 560 nm, which is proportional to the total antioxidant capacity. A standard curve from the ferrous chloride (FeCl₂) solution allows researchers to determine antioxidant capacity by measuring absorbance values. The TAC concentration was calculated from a standard curve. The curve was obtained by plotting the ΔOD values of standard solutions versus their respective concentrations^32^.

#### Caspase-3 expression analysis

The experimental design included four distinct groups where cells received (a) no treatment (0 h baseline), (b) exposure to 10 µM Aβ, (c) 10 µM free GRg3 + 10 µM Aβ, and (d) 10 µM PEGylated GRg3-loaded niosome + 10 µM Aβ following 6, 12, and 24 h. Total RNA was extracted from neuroblastoma cells using a Sinaclon extraction kit (Tehran, Iran) before performing reverse transcription synthesis of cDNA from 1 µg RNA according to Pars Tous (Iran) manufacturer instructions. *Caspase-3* mRNA levels were quantified using specific primers (forward: 5′-TGTCATCTCGCTCTGGTACG-3′, reverse: 5′-AAATGACCCCTTCATCACCA-3′)^33^ normalized to the housekeeping gene *GAPDH* (forward: 5′-GAGCCATCGTCAGACAC-3′, reverse: 5′-CATGTAGTTGGAATGAAGG-3′)^34^. Relative *caspase-3* expression was determined using the 2^−ΔΔCT^ method.

### In vivo studies

#### Animals and experimental design

A total number of 40 adult male Wistar rats (weighing 200–250 g) were obtained from the institutional animal facility and housed under standard laboratory conditions (12 h light/dark cycle, 22 ± 2 °C, 50–60% humidity) with ad libitum access to food and water. Animal care and handling complied with NIH regulations, Iranian governmental licensing requirements for animal experimentation, and the ARRIVE guidelines (accessible at https://arriveguidelines.org) for the full and transparent reporting of research involving animals.

On day 8, memory impairment was induced by bilateral intracerebroventricular (ICV) administration of aggregated amyloid-β (Aβ1–42). The peptide was dissolved in sterile distilled water and incubated at 37 °C for 4–7 days to promote aggregation. Following anesthesia with ketamine (80 mg/kg) and xylazine (10 mg/kg), 5 µL of Aβ (5 µg/µL per side) was carefully delivered into the lateral ventricles at defined coordinates relative to the bregma (AP − 0.8 mm, ML ± 1.5 mm, DV − 3.6 mm) under aseptic conditions. Control animals received an equivalent volume of PBS. The experimental subjects were divided into several groups. The control group received intracerebroventricular (ICV) injections of sterile PBS, along with daily intraperitoneal (i.p.) injections of saline at a dose of 1 mL/kg. The Aβ group was administered ICV injections of Aβ without any subsequent treatment. The Aβ + GRg3 group received Aβ via ICV injection and was subjected to daily i.p. injections of free GRg3 at a dosage of 10 mg/kg, dissolved in saline. Lastly, the Aβ + PEGylated GRg3-loaded niosome group also received Aβ via ICV injection and was treated daily with i.p. injections of PEGylated GRg3-loaded niosome containing an equivalent of 10 mg/kg of GRg3 in saline. All interventions were maintained for three weeks following the Aβ injection. Behavioral tests (such as Morris Water Maze or Novel Object Recognition) were performed in the final week to assess memory function and confirm cognitive impairment.

At the end of the experimental period, animals were anesthetized with ketamine and xylazine and euthanized by decapitation under deep anesthesia. Brains were removed immediately and divided in the sagittal plane. One hemisphere was rinsed in ice-cold PBS, snap-frozen in liquid nitrogen, and stored at − 80 °C for biochemical analyses (e.g., oxidative stress markers, antioxidant enzymes). The other hemisphere was processed for histopathological and immunohistochemical analysis^35^.

#### Assessment of Spatial memory and cognition in the AD model

The Y-maze consisted of three identical opaque arms (A, B, C) made of black Plexiglas, each measuring 46 cm (length) × 15 cm (width) × 15 cm (height), positioned at 120° angles from each other. The maze was elevated 50 cm above the floor and placed in a dimly lit, sound-attenuated room to minimize external stress. Habituation: Each rodent was placed at the end of one arm (randomly selected) and allowed to explore freely for 2 min while confined to that arm^4^. After habituation, the animal was granted access to all arms for 6 min, and its movements were recorded using a video tracking system. An arm entry was counted only when the entire body, including the tail, crossed into the arm. After each test, the chamber was cleaned using a 90% alcohol solution.

#### Open field test (OFT) setup

The Open Field Test (OFT) was performed in rats to assess spatial memory through habituation-based memory, object-in-context memory, and spatial navigation. The OFT arena was a 100 cm × 100 cm × 40 cm wooden enclosure with white walls and floor. It had a central zone (25% of the total area: 50 cm × 50 cm) and peripheral zones marked by painted lines. The arena was cleaned with 70% ethanol between trials to remove odor cues. Rats were acclimated to the testing room for 1 h before the experiments. Each rat was placed in the center of the arena and observed for 10 min by two trained, blinded observers. The time spent in the central zone, with all four paws inside, was recorded using a stopwatch. Locomotor activity (e.g., freezing, rearing) was qualitatively noted. Observer recordings were cross-verified for consistency. The time spent in the central zone was presented as mean ± SEM.

#### Three-chamber tests

The sociability assessment was conducted using three-chamber tests (an apparatus with three equal chambers, 70 cm wide and 30 cm long). Before the beginning of the primary phase, the offspring were placed in a central chamber for a 5-minute habituation. After habituation, the sociability phase was started and lasted for 10 min. Each animal had free access to side chambers. The time spent in each of the three chambers (center, animal chamber, and empty-cage chamber) was recorded by a camera. After each test, all three chambers were cleaned using a 90% alcohol solution.

#### Morris water maze (MWM) apparatus

During the experiment, control rats underwent a habituation phase to acclimatize them to the swimming pool, where each rat experienced 60 s of free swimming daily without a platform. This approach aimed to minimize stress and help the rats adapt to their new environment. Following habituation, the acquisition phase focused on spatial learning, where the rats participated in four trials each day for four consecutive days. Each trial began from pseudorandomized starting positions to eliminate bias and concluded when a rat successfully located the platform within 60 s or was guided to it if unsuccessful. Escape latency was carefully recorded, and trials in which rats spent more than 50% of their time in the pool’s periphery or exhibited freezing behavior were excluded from further analysis.

In the following probe test, we removed the platform and gave the rats a 60-second free-swim trial starting from a new quadrant to evaluate their spatial memory. Key metrics included the time spent in the target quadrant and the number of times rats crossed within a 10 cm radius of the platform’s former location. To maintain the integrity of the study, environmental and behavioral controls were implemented, including consistent lighting, a water temperature of 22 ± 1 °C, and regulated noise levels.

#### Immunohistochemistry and histopathology

Transverse brain Sect. (5 μm thick) were prepared from paraffin-embedded tissue blocks. For histopathological evaluation, sections were stained with hematoxylin and eosin (H&E) and examined under an optical microscope (Olympus, Tokyo, Japan). Transverse brain Sect. (5 μm) prepared from paraffin-embedded mouse brain tissue were immunohistochemically stained using the glial fibrillary acidic protein (GFAP) marker. The primary antibody was provided in the GFAP kit (Cat. No. PMD-008; Diagnostic BioSystems, USA). For visualization, a secondary antibody was applied using the immunohistochemistry kit from Vitro Master Diagnostica (Spain), according to the manufacturer’s protocol. Stained sections were analyzed by optical microscopy.

#### Biochemical assays

##### Superoxide dismutase (SOD) activity

SOD activity in the brain tissue of adult male rats was measured using the nitroblue tetrazolium (NBT) reduction assay. Brain regions were dissected, homogenized in ice-cold PBS, and centrifuged (10,000 × g, 15 min) to obtain supernatants. SOD activity was assessed spectrophotometrically at 560 nm using a reaction mixture containing sodium carbonate buffer (pH 10.2), EDTA, xanthine, NBT, and xanthine oxidase, with activity expressed as units/mg protein (one unit inhibits 50% NBT reduction). The protocol adhered to foundational methods with minor changes (Beauchamp & Fridovich, 1971; Bradford, 1976).

##### Brain catalase

Brain tissue samples were homogenized in ice-cold 50 mM phosphate buffer (pH 7.0, 1:5–1:10 w/v ratio) using a mechanical homogenizer, followed by centrifugation at 10,000 × g (4 °C, 20 min) to isolate supernatants. Catalase activity (CAT) was measured via the Aebi et al. method with minor modifications^36^.

##### Brain MDA levels

Brain MDA levels were measured using the thiobarbituric acid reactive substances (TBARS) assay, following the method of Ohkawa et al^37^., with minor modifications. Briefly, brain tissue was homogenized in ice-cold PBS and centrifuged at 10,000 × g for 10 min at 4 °C. The supernatant was mixed with 0.67% thiobarbituric acid (TBA) and 10% trichloroacetic acid (TCA), and then incubated at 95 °C for 15 min. After cooling, the mixture was centrifuged, and the absorbance of the supernatant was measured at 532 nm using a spectrophotometer.

### In Silico studies

In this study, MD simulations were conducted to investigate the self-assembly behavior of a niosomal bilayer composed of T40, S40, CHL, and GRg3. Initially, the molecular structures of these components were constructed and optimized using Gaussian^38^ at the B3LYP/6–311 + + G(d, p) level of theory.​ To model the self-assembly process, a simulation box measuring 200 × 200 × 200 Å³ was prepared, containing 350 molecules of T40 and S40, 300 molecules of CHL, 5 molecules of GRg3, and approximately 40,000 water molecules. The initial configuration was generated using the Packmol software^39^, ensuring a random and homogeneous distribution of all components within the simulation box.​.

Force field parameters for T40, S40, and CHL were derived from the Lipid21 force field^40^ within the AMBER framework, which is suitable for simulating complex lipid membranes. GRg3 parameters were assigned using the General Amber Force Field (GAFF)^49^, which is appropriate for small organic molecules. The system was then converted to a GROMACS-compatible format for simulation.​ Energy minimization was performed using the steepest descent algorithm to eliminate unfavorable contacts. Subsequently, a short annealing simulation at 500 K for 100 ps was conducted to resolve any remaining steric clashes. The system was then equilibrated under the NVT ensemble at 300 K for 500 ps using the V-rescale thermostat with a coupling constant of 0.1 ps. This temperature was chosen based on previous studies indicating optimal stability of niosomal vesicles under this condition.​ Following temperature equilibration, the system underwent NPT equilibration for 3 ns at 300 K and 1 bar pressure, employing the Parrinello-Rahman barostat with isotropic coupling. Periodic boundary conditions were applied in all three dimensions. Bond constraints were enforced using the LINCS algorithm^41^, and long-range electrostatic interactions were calculated using the Particle Mesh Ewald (PME) method. A cutoff of 1.4 nm was set for both Coulombic and van der Waals interactions.​Finally, a production MD simulation was carried out for 100 ns using GROMACS version 2024.2^42^. Trajectory data were collected for subsequent analysis of bilayer properties and the distribution of GRg3 within the membrane.

### Statistical analysis

All data are expressed as mean ± SD. Statistical analyses were conducted using Student’s t-test and one-way ANOVA in GraphPad Prism 9, with a significance threshold set at *p* < 0.05. Additionally, two-way ANOVA followed by post-hoc analysis using the Tukey test was performed. During the acquisition phase of the Morris water maze test, learning metrics, including escape latency and path length, were averaged daily for each subject. Data are presented as mean ± SD from independent experiments conducted in triplicate.

## Results

### Physicochemical characterization

#### Morphology, size, and zeta potential assessments

**Fig. 1:**
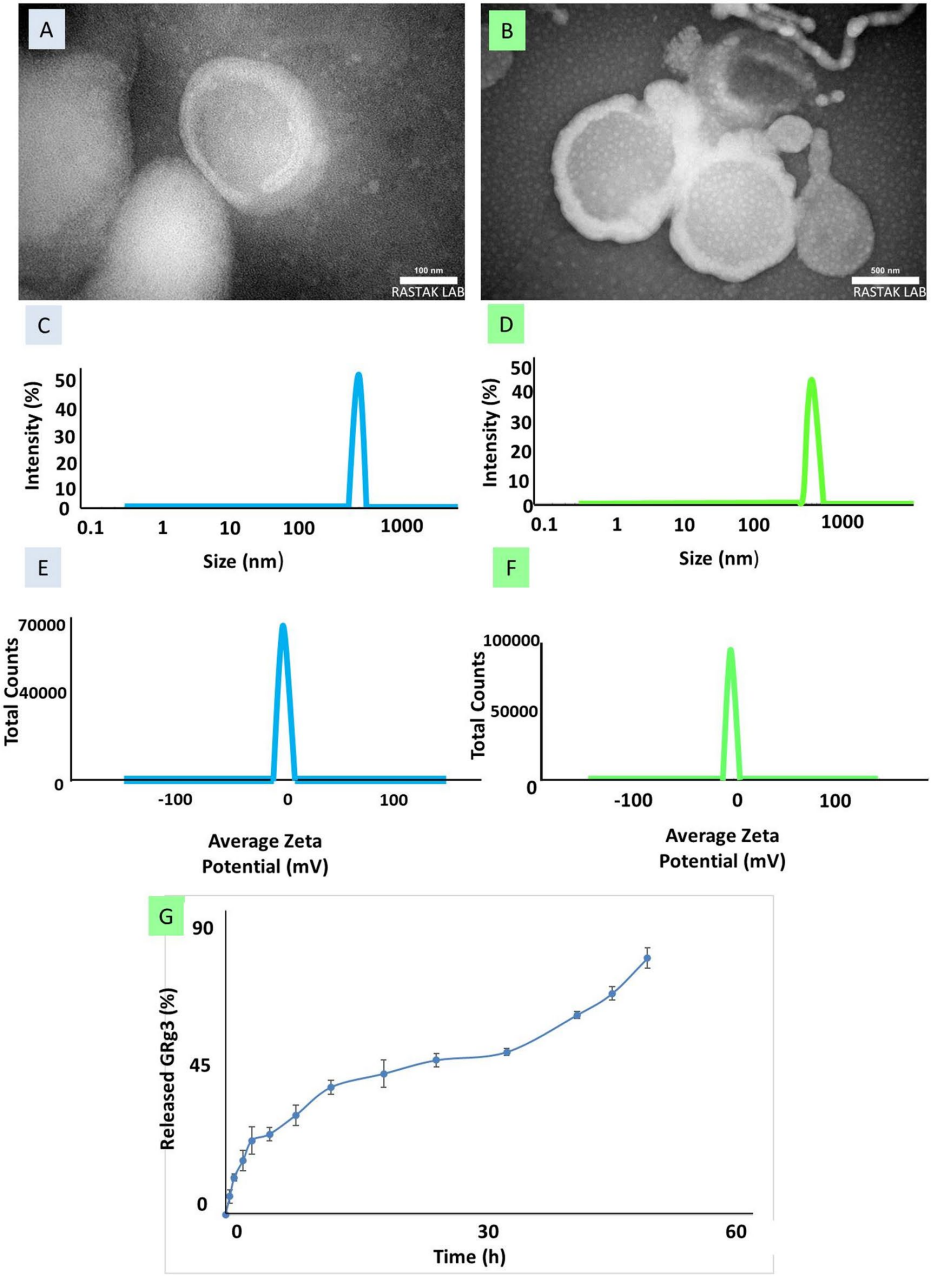
**(A)** TEM images of GRg3-loaded noisome, **(B)** TEM images of PEGylated GRg3-loaded noisome, **(C)** hydrodynamic diameter of GRg3-loaded noisome, **(D)** hydrodynamic diameter of PEGylated GRg3-loaded noisome, **(E)** zeta potential (mV) of GRg3-loaded noisome, **(F)** zeta potential (mV) of PEGylated GRg3-loaded noisome and **(G)** cumulative in vitro release profile of GRg3 from PEGylated GRg3-loaded noisome in PBS (pH 7.4) at 37 °C within 48 h.

The TEM image of the GRg3-loaded noisome revealed small, spherical vesicles with well-defined borders. These vesicles exhibited a relatively consistent morphology, and the bilayer membrane structure was discernible. Given the 100 nm scale bar, the particle sizes appeared to be mostly within the 100–200 nm range. The image showed discrete, sharply contoured vesicles without any visible surface coating (Fig. 1A).

In the PEGylated GRg3-loaded noisome image, vesicles maintained a spherical morphology but appeared larger in diameter relative to the non-PEGylated group. Based on the 500 nm scale bar, the diameter of many vesicles ranged from approximately 500 to 800 nm. The borders of these vesicles appeared less sharp or more diffuse compared to the GRg3-loaded noisome, which is a typical visual signature of a hydrated polymer shell (Fig. 1B). This observation indicated the presence of a PEG layer surrounding the vesicles. DLS analysis showed that the GRg3-loaded noisome formulation had a particle size distribution centered around 600–700 nm, indicating relatively uniform vesicle formation (Fig. 1C). In comparison, the PEGylated formulation, PEGylated GRg3-loaded noisome (B), exhibited a larger particle size range, with peaks around 800–900 nm, reflecting an increase in hydrodynamic diameter due to PEG modification (Fig. 1D). Zeta potential measurements revealed that GRg3-loaded noisome had a zeta potential of −3.56 mV, while the PEGylated GRg3-loaded noisome showed a more negative surface charge (−5.30 mV) (Fig. 1E and F).

#### Drug loading efficiency and release

The EE was calculated to be approximately 83.02%. This indicated that most of the GRg3 was successfully encapsulated within the PEGylated GRg3-loaded noisome, demonstrating the formulation’s effectiveness in drug entrapment. For the in vitro drug release study, the drug release was assessed in PBS (pH 7.4) over 48 h using the dialysis method. No drug release was detected at the initial time point (0 h). Within the first 30 min, approximately 5.33% of the drug was released, which increased to 10.93% after one h. The release continued to increase over time, reaching 29.33% at 8 h and 37.6% at 12 h. By 24 h, 45.6% of the drug had diffused into the external medium, and at the final time point (48 h), the cumulative drug release reached 75.73%, indicating a sustained release profile. The gradual release pattern suggested that PEGylated GRg3-loaded noisome effectively controlled the release of GRg3, which could be beneficial for prolonged drug availability and reduced dosing frequency (Fig. 1G). Based on our findings, the high EE and sustained drug release profile demonstrated the potential of PEGylated GRg3-loaded noisome as an efficient delivery system for GRg3.

When the data were fitted to the different kinetic models, the Korsmeyer-Peppas model demonstrated the best correlation with an R² value of 0.982. This was followed by the Higuchi model (R² = 0.965), zero-order (R² = 0.954), Hixson-Crowell (R² = 0.948), and first-order (R² = 0.931) models. The release exponent (n) derived from the Korsmeyer-Peppas model was found to be 0.447. This value indicated that the drug release followed Fickian diffusion, where the rate-controlling step is primarily governed by diffusion through the polymer matrix, rather than by erosion or swelling mechanisms. These findings suggested that the PEGylated GRg3-loaded noisome enabled a controlled and diffusion-driven release of the encapsulated drug, making it potentially suitable for sustained-release therapeutic applications.

###  In-vitro assays

#### MTT assay

Results from MTT assays indicated that Aβ exposure led to significant decreases in neuroblastoma cell viability. The addition of GRg3 or PEGylated GRg3-loaded noisome during co-treatment showed protective effects against Aβ-induced cytotoxicity, leading to improved cellular viability compared to the Aβ-treated cells. Free GRg3 at 10 µM and all concentrations of PEGylated GRg3-loaded noisome, ranging from 5 µM to 20 µM, restored viability to match that of the untreated control levels (Table 1). In this study, treatment with Aβ alone resulted in only 51% cell viability, indicating significant cytotoxicity. However, co-treatment with either free GRg3 or PEGylated GRg3-loaded niosomes significantly improved cell viability. Notably, no consistent dose-dependent trend was observed between increasing drug concentrations and cell viability for either formulation within the tested range. Nonetheless, the highest viability rates were observed at a concentration of 10 µmol/L, reaching 91% for free GRg3 and 98% for the PEGylated GRg3-loaded niosomes. Consequently, this concentration was selected for subsequent experiments.

**Table 1:** Viability percentages of SH-SY5Y cells were treated with either Aβ alone or in combination with increasing concentrations of free-GRg3 or pegylated GRg3-loaded noisome. Statistical significance was determined as follows: *p < 0.05 compared to the control group, and #*p* < 0.05 compared to the Aβ (10 µmol/L) treatment group.

Concentration in Treatment Groups				Cell Viability (%)
Control (DMSO, v/v %)	Free GRg3 (µmol/L)	PEGylated GRg3-loaded noisome (µmol/L)	Aβ (µmol/L)	
1	-	-	-	100
-	-	-	10	51.33 ± 3.09
-	2.5	-	10	73.21 ± 5.72^*#^
-	5	-	10	79.57 ± 4.85^*#^
-	10	-	10	91.66 ± 2.41^#^
-	20	-	10	88.99 ± 6.66^#^
-	40	-	10	81.07 ± 4.19^*#^
-	-	2.5	10	80.19 ± 3.07^*#^
-	-	5	10	92.71 ± 1.96^#^
-	-	10	10	98.06 ± 0.72^#^
-	-	20	10	90.51 ± 2.79^#^
-	-	40	10	82.39 ± 3.48^*#^

#### In vitro lipid peroxidation and antioxidant capacity

**Fig. 2:**
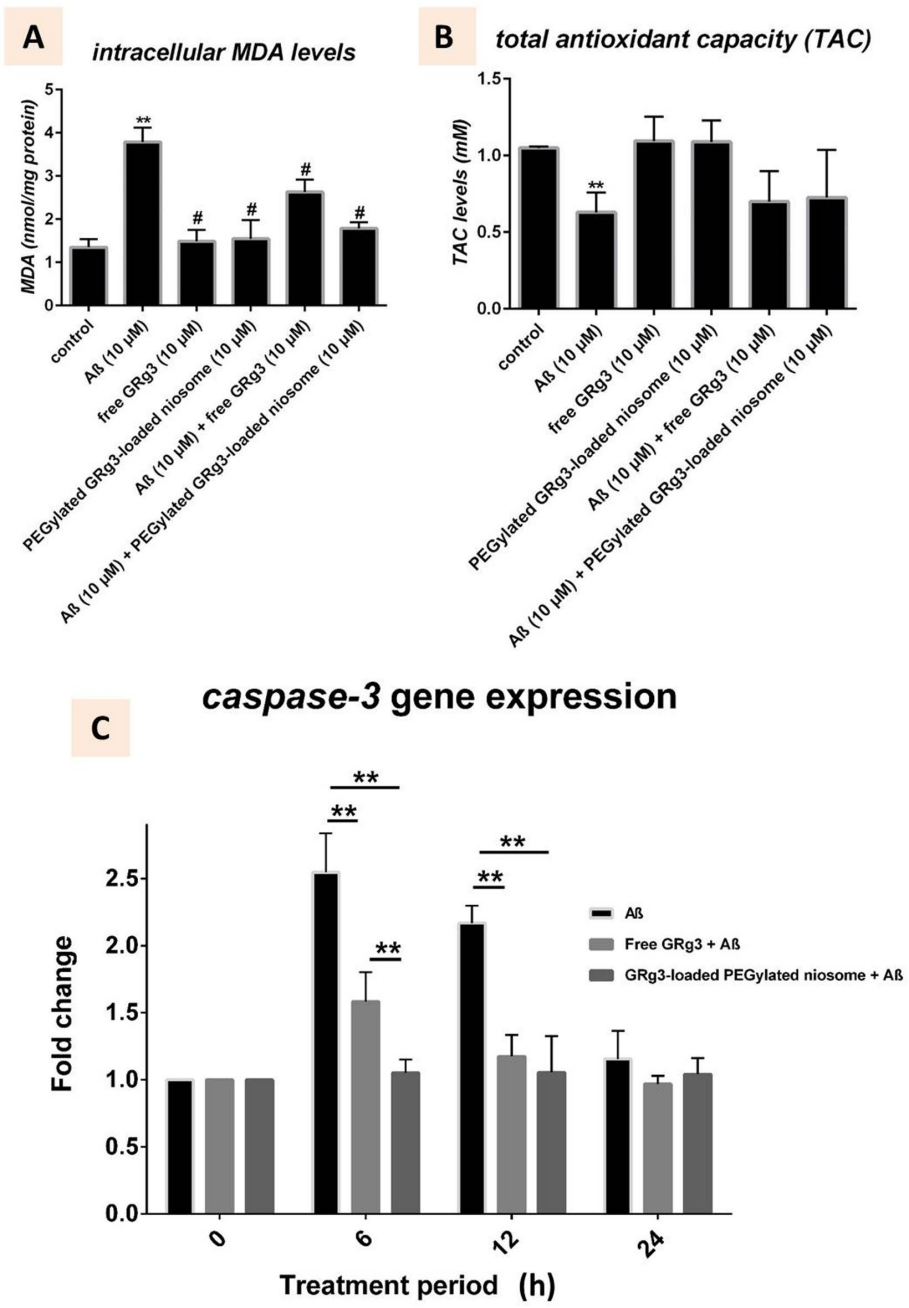
**(A)** Both free and PEGylated GRg3-loaded niosomes suppressed Aβ-induced oxidative stress damage, as shown by the decrease in MDA synthesis compared to the Aβ-treated group (*p* < 0.05, *n* = 3). **Compared to the control group and # compared to Aβ-treated group, **(B)** The TAC measurements in Aβ-treated SH-SY5Y Cells receiving free and PEGylated GRg3-loaded niosome, **(C)** Assessment of the relative *caspase-3* mRNA expression levels normalized to *GAPDH* in Aβ (10 µM)-treated neuroblastoma cell lines with free GRg3 (10 µM) and PEGylated GRg3-loaded niosome (10 µM). Compared to the control group. **p < 0.05 indicates a significant difference compared to the control group.

As indicated in Fig. 2A, the intracellular levels of MDA in the Aβ-treated group were drastically higher (1.35 ± 0.19 nmol/mg) than those of the control group (3.79 ± 0.33 nmol/mg protein; *p* < 0.05, *n* = 3). However, co-treatment with free and PEGylated GRg3-loaded noisome, along with Aβ, markedly mitigated the elevation of MDA levels, lowering them to 2.63 ± 0.29 and 1.79 ± 0.14 nmol/mg protein, respectively, compared with the Aβ-treated group (*p* < 0.05, *n* = 3).

The experimental results presented in Fig. 2B show that exposure of SH-SY5Y cells to 10 µM Aβ resulted in a significant reduction of TAC levels compared to controls (*p* < 0.05). The TAC levels in cells treated with Aβ (10 µM) remained statistically similar to cells exposed to Aβ (10 µM) co-administered with free GRg3 (10 µM) or co-administered with PEGylated GRg3-loaded noisome (10 µM) (1.06 ± 0.03 vs. Aβ-treated group; *p* > 0.05 for both comparisons). The study results indicate that GRg3 exhibits intrinsic antioxidant properties in neuroblastoma cells. Still, its free form and niosomal formulation fail to recover Aβ-impaired antioxidant capacity in the examined experimental conditions.

#### Caspase-3 expression analysis

The gene expression level of *caspase-3* increased significantly in the Aβ-treated group during the 6 h and 12 h observation periods, reaching 2.5-fold and 2.0-fold increases relative to baseline measurements at 0 h, as shown in Fig. 2C. The expression levels of *caspase-3* showed limited increases when cells received free GRg3 treatment at 1.7-fold during 6 h and 1.3-fold during 12 h. Yet, the PEGylated GRg3-loaded noisome maintained the lowest *caspase-3* expression at 1.2-fold at both time points. The results confirm that both GRg3 formulations showed significant differences against Aβ exposure at 6 and 12 h (*p* < 0.05), demonstrating that GRg3 and its niosomal delivery system can reduce Aβ-induced apoptotic signaling. The Aβ-treated group exhibited *caspase-3* expression levels that returned towards baseline by 24 h without significant variations between the free GRg3 and PEGylated GRg3-loaded noisome at this time. The data indicate that the Aβ peptide activates caspase-3 intensely but briefly during the initial 12 h, during which GRg3 is successfully decreased. The niosomal formulation delivered GRg3 with superior effectiveness in reducing early apoptotic events compared to free GRg3 treatment of Aβ exposure.

### In vivo results

#### Cognitive and behavioral outcomes

##### Y-Maze test

**Fig. 3:**
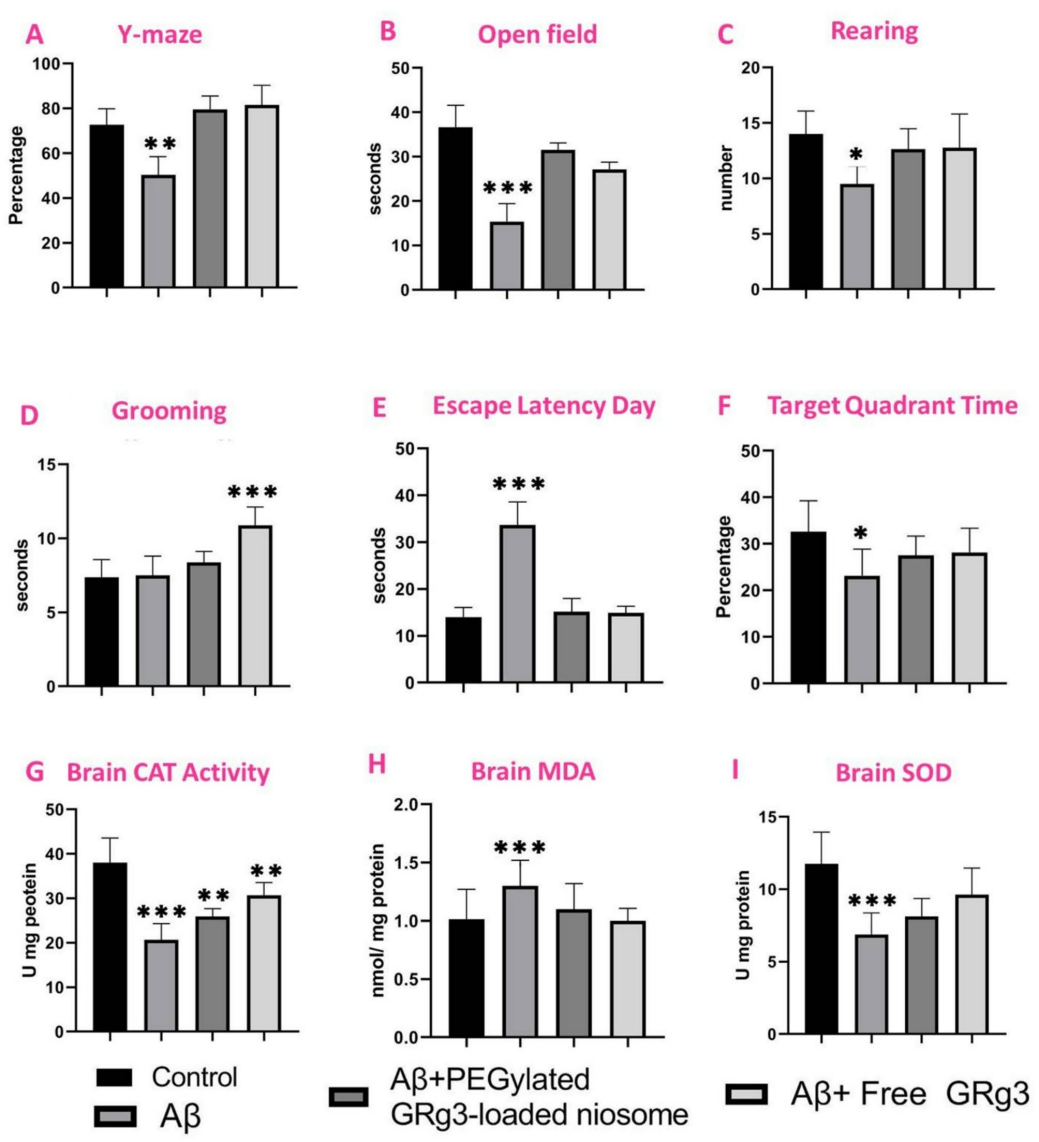
Behavioral performance in rats: **(A)** alternation score (%) in the Y-maze test, reflecting the memory function, **(B)** time spent in the center (s) of the open field test, indicative of anxiety-like behavior, **(C)** rearing frequency (count) and **(D)** grooming duration (s) recorded during the three-chamber test. Morris water maze performance: **(E)** escape latency (s) and **(F)** target quadrant time (%). Brain oxidative stress markers in experimental groups: **(G)** catalase activity, **(H)** MDA levels, and **(I)** SOD activity in brain tissue. * Indicates a statistically significant difference compared to the control group (*p* < 0.05). **** ** Indicates a significant difference (*p* < 0.01). ****** **** ** Indicates a significant difference (*p* < 0.001).

The Y-Maze alternation test revealed significant impairments in spatial working memory in the Aβ-treated group (T2), which showed a 30.6% reduction in alternation scores compared to controls (T1) (*p* < 0.0001). Treatment with PEGylated GRg3-loaded noisome (T3) and free GRg3 (T4) effectively restored alternation scores to control levels (*p* < 0.0001 vs. T2), with no significant difference between the two treatment groups (*p* = 0.959). These findings suggest that both niosomal formulation and free GRg3 effectively reverse Aβ-induced cognitive deficits, with comparable therapeutic efficacy (Fig. 3A).

##### The OFT

Control animals treated with saline spent significantly more time in the center of the open field (36.63 ± 4.98 s) compared to untreated AD rats (15.4 ± 4.1 s; *p* < 0.001), indicating Aβ-induced AD markedly increased anxiety-like behavior. AD rats administered PEGylated GRg3-loaded noisome (31.5 ± 1.6 s) exhibited a significant restoration of exploratory behavior relative to untreated diabetic rats (*p* < 0.001), with time spent in the center approaching control levels. Similarly, free GRg3 treatment (27.13 ± 1.6 s) partially ameliorated anxiety-like behavior compared to T2 (*p* < 0.001), although its efficacy was inferior to that of PEGylated GRg3-loaded noisome.

Notably, the PEGylated GRg3-loaded noisome demonstrated superior therapeutic potential over free GRg3, as evidenced by the near-normalization of exploratory behavior in T3 (no significant difference vs. T1 at the *p* < 0.01 threshold). These findings suggest that PEGylation enhances the efficacy of GRg3 in mitigating AD-associated anxiety-like behaviors. However, the difference between PEGylated GRg3-loaded noisome and free GRg3 was insignificant at the *p* < 0.05 threshold (Fig. 3B).

#### Three-chamber behavioral assessment

##### Rearing behavior

Rearing activity varied significantly across experimental groups. Control animals exhibited a mean rearing frequency of (14 ± 2). In contrast, the Aβ-treated group demonstrated a marked reduction in rearing behavior (9.5 ± 1.6); *p* < 0.05 vs. control. Rats treated with PEGylated GRg3-loaded noisome (10 mg/kg, i.p.) showed partial restoration of rearing activity (12.62 ± 1.8); *p* < 0.05 vs. Aβ-treated group, though values remained below control levels (*p* > 0.05 vs. control). Similarly, rats administered free GRg3 (10 mg/kg, i.p.) displayed improved rearing (12.75 ± 3.0), with significant differences compared to untreated rats (*p* < 0.05) and a marginal but statistically non-significant difference relative to the PEGylated GRg3-loaded noisome group (Fig. 3C).

##### Grooming behavior

Grooming frequency in control (7.3 ± 1.18) and Aβ-treated group (7.5 ± 1.3) did not differ significantly (*p* > 0.05). Treatment with PEGylated GRg3-loaded niosome resulted in a non-significant increase in grooming activity (8.3 ± 0.74); *p* > 0.05 vs. control and Aβ-treated group. Strikingly, rats administered free GRg3 exhibited a pronounced elevation in grooming behavior (10.8 ± 1.2), which significantly exceeded that of all other groups, including controls (*p* < 0.001) (Fig. 3D). These findings suggest that beta amyloid impairs rearing behavior, which is partially rescued by both free and nanoformulated GRg3. However, free GRg3 demonstrates superior efficacy in ameliorating rearing deficits and uniquely enhances grooming behavior beyond physiological levels observed in healthy controls. The differential effects highlight the potential influence of drug formulation on therapeutic outcomes.

#### Morris water maze

Morris water maze testing revealed significant cognitive deficits in the Aβ-treated group compared to the healthy control group (Figs. 3E and F). Rats exhibited a 59.3% increase in escape latency compared to controls (*p* < 0.05), indicating impaired spatial learning. Furthermore, target quadrant time, a measure of memory retention, was significantly reduced in Aβ rats (*p* < 0.05), with healthy rats spending 29.6% more time in the target quadrant. Administration of PEGylated GRg3-loaded niosome (10 mg/kg) significantly improved spatial learning in rats. Escape latency in this group was markedly reduced compared to the Aβ-treated group (*p* < 0.001), representing a 55.0% decrease. However, target quadrant time in the PEGylated GRg3-loaded niosomes group showed only a non-significant increase relative to the Aβ-treated group (*p* > 0.05). Daily treatment with free GRg3 (10 mg/kg) similarly reduced escape latency in Aβ rats (14.8 vs. 33.6 in Aβ-treated group; 56.0% decrease), though this difference did not reach statistical significance (*p* > 0.05). Target quadrant time in the free GRg3 group was also modestly elevated compared to the Aβ-treated group, but this improvement was insignificant (*p* > 0.05). Both free GRg3and PEGylated GRg3-loaded niosome partially reversed cognitive deficits, with free GRg3 demonstrating marginally more significant numerical improvements in target quadrant time (28.1 vs. 27.5 for PEGylated GRg3-loaded niosome). However, only PEGylated GRg3-loaded niosomes achieved a statistically significant reduction in escape latency (*p* < 0.001), highlighting enhanced efficacy in spatial learning restoration. These findings demonstrate that Aβ treatment significantly impairs spatial learning and memory, while PEGylated GRg3-loaded niosomes elicit a robust improvement in spatial learning, as evidenced by reduced escape latency. Both formulations showed trends toward restoring memory retention, though further studies are warranted to optimize therapeutic outcomes.

#### Effects of GRg3 formulations on brain oxidative stress markers

##### Catalase (CAT) activity

The effects of PEGylated GRg3-loaded niosome and free GRg3 on CAT activity were examined in the Aβ-treated group (Fig. 3G). The control group maintained normal CAT activity levels (36.20 ± 5.00 U/mg protein), while the Aβ-treated group exhibited significantly reduced CAT activity (20.63 ± 3.70 U/mg protein; *p* < 0.001 vs. control), indicating substantial impairment of antioxidant defenses. Treatment with PEGylated GRg3-loaded niosome demonstrated superior efficacy in restoring CAT activity (30.63 ± 2.90 U/mg protein) compared with free GRg3 treatment (25.88 ± 1.81 U/mg protein). Notably, the PEGylated GRg3-loaded niosome group showed higher CAT activity than the free GRg3 group, suggesting enhanced therapeutic effects of the niosomal formulation.

##### MDA levels

The effects of PEGylated GRg3-loaded niosomes and free GRg3 on MDA levels, a marker of LPO, were evaluated in Aβ-injected rats (Fig. 3H). Four experimental groups were compared: control (T1), Aβ-injected untreated (T2), Aβ-injected treated with PEGylated GRg3-loaded niosome (T3), and Aβ-injected treated with free GRg3 (T4). The control group (T1) showed baseline MDA levels of 1.01 ± 0.26 nmol/mg protein. As expected, the Aβ-injected group without treatment (T2) exhibited significantly elevated MDA levels (1.30 ± 0.20 nmol/mg protein), indicating increased oxidative stress (*p* = 0.048 vs. control). Treatment with PEGylated GRg3-loaded niosome (T3) reduced MDA levels to 1.10 ± 0.22 nmol/mg protein, while free GRg3 treatment (T4) showed the most pronounced effect, lowering MDA to control levels (1.00 ± 0.11 nmol/mg protein). Post-hoc analysis revealed that free GRg3 treatment (T4) significantly reduced MDA levels compared to the Aβ-injected group (T2; *p* = 0.037). However, the difference between PEGylated GRg3-loaded niosome (T3) and the Aβ-injected group (T2) did not reach statistical significance (*p* = 0.247). Importantly, MDA levels in both treatment groups (T3 and T4) showed no significant difference from control values (T1; *p* = 0.837 and *p* = 0.999, respectively).

##### SOD activity

The effects of PEGylated GRg3-loaded niosomes and free GRg3 on SOD activity were evaluated in rats and compared with control and Aβ-treated group (Fig. 3I). The control group (T1), receiving daily i.p. injections of sterile saline, exhibited the highest SOD activity (11.7 ± 2.19). In contrast, the Aβ-treated group (T2) showed a significant reduction in SOD levels (6.9 ± 1.5), confirming Aβ-induced oxidative stress. Treatment with PEGylated GRg3-loaded niosomes (T3) partially restored SOD activity (8.1 ± 1.2), whereas free GRg3 administration (T4) resulted in more substantial recovery (9.6 ± 1.8). Post-hoc analysis using Tukey’s HSD test revealed significantly lower SOD activity in the Aβ-treated group (T2) compared to the controls (T1) (*p* < 0.001). While both treatments improved SOD levels relative to the Aβ group, only free GRg3 (T4) showed a statistically significant increase versus the Aβ-treated group (*p* = 0.017). Neither treatment restored SOD to control levels. The difference between PEGylated GRg3-loaded niosomes (T3) and free GRg3 (T4) was not statistically significant (*p* = 0.322), suggesting comparable efficacy. Free GRg3 (T4) showed a non-significant trend toward higher SOD activity versus controls (T1) (*p* = 0.088).

#### Histopathological analysis

**Fig. 4:**
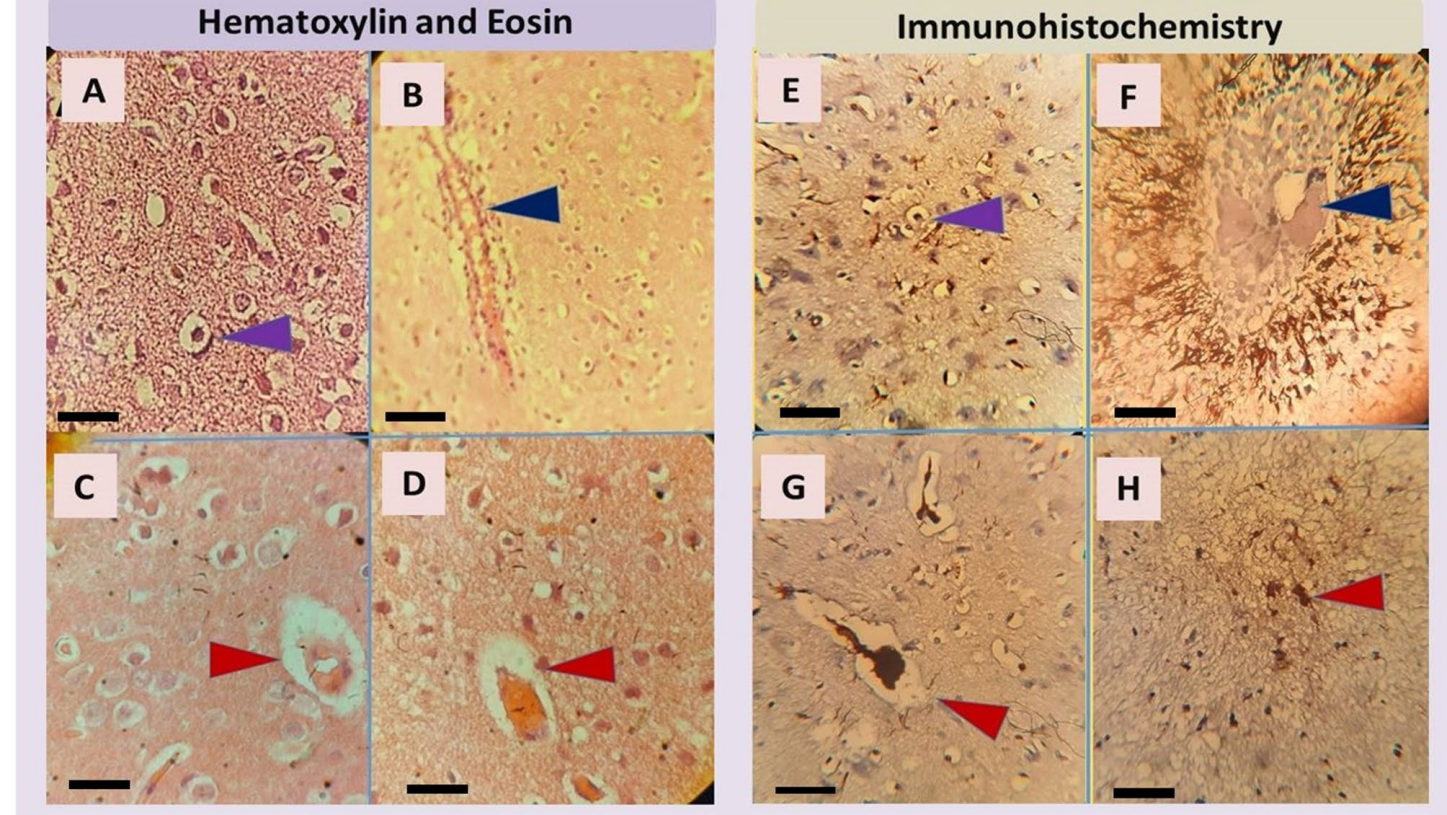
Representative H&E-stained sections of hippocampal brain tissue from experimental groups: (A) control group showing normal histological structure with preserved microvasculature, (B) Aβ-treated group showing mild vascular alterations indicative of early microvascular dysfunction, (C) Aβ + PEGylated GRg3-loaded niosome group exhibiting partial improvement in vascular morphology, (D) Aβ + free GRg3 group demonstrating near-complete preservation of vascular integrity with minimal changes. Immunohistochemically-stained sections of hippocampal brain tissue from experimental groups: (E) control group, normal hippocampal architecture with no detectable Aβ plaque deposition, (F) Aβ-treated group (pronounced accumulation of Aβ plaques, indicative of early AD pathology). (G) Aβ + PEGylated GRg3-niosomes group (enlargement of the nucleus and reduced dendritic size in osteocytes, indicating improved tissue morphology and partial preservation of cellular integrity), (H) Aβ + free GRg3 group: Increased perineuronal space, suggestive of ongoing neuronal stress or damage despite treatment. The scale bar indicates 20 μm.

Hematoxylin and eosin (H&E) staining of brain sections from the control group was normal (Fig. 4A). In contrast, the Aβ-treated group revealed mild microvascular alterations, including microhemorrhages and microvascular rarefaction, indicative of BBB dysfunction (Fig. 4B). In contrast, brain sections from rats treated with PEGylated GRg3-loaded niosomes exhibited preserved microvascular integrity, with no signs of microhemorrhages or endothelial disruption. Neuronal morphology appeared intact, with well-defined nuclei and preserved cytoarchitecture, indicating a protective effect against neurovascular damage (Fig. 4C). Similarly, rats treated with free GRg3 showed no significant histopathological abnormalities, maintaining a normal brain structure. Neurons were well-preserved, and no signs of gliosis, microvascular damage, or neurodegeneration were detected (Fig. 4D). These findings suggest that both PEGylated GRg3-loaded niosomes and free GRg3 treatment effectively mitigated BBB dysfunction and neurovascular damage, with PEGylated GRg3-loaded niosomes demonstrating comparable neuroprotective efficacy to free GRg3.

#### Immunohistochemical analysis

Immunohistochemical analysis of hippocampal brain sections revealed distinct histopathological differences across treatment groups. In the control group, normal hippocampal cytoarchitecture was maintained, with no detectable Aβ plaques. In contrast, the Aβ group displayed substantial Aβ deposition and disrupted tissue architecture, consistent with early AD pathology. Notably, treatment with PEGylated GRg3-loaded niosome (Aβ + PEGylated GRg3-niosomes group) resulted in visible improvements in tissue morphology, characterized by nuclear enlargement and reduced dendritic atrophy in osteocytes, suggesting partial preservation of cellular integrity. However, administration of free GRg3 (Aβ + free GRg3 group) was associated with increased perineuronal space, indicative of persistent neuronal stress or incomplete neuroprotection. These findings suggest that PEGylated GRg3-loaded niosomes offer enhanced therapeutic benefits over the free compound in mitigating Aβ-induced hippocampal damage (Fig. 4E-H).

### MD simulation analysis

**Fig. 5:**
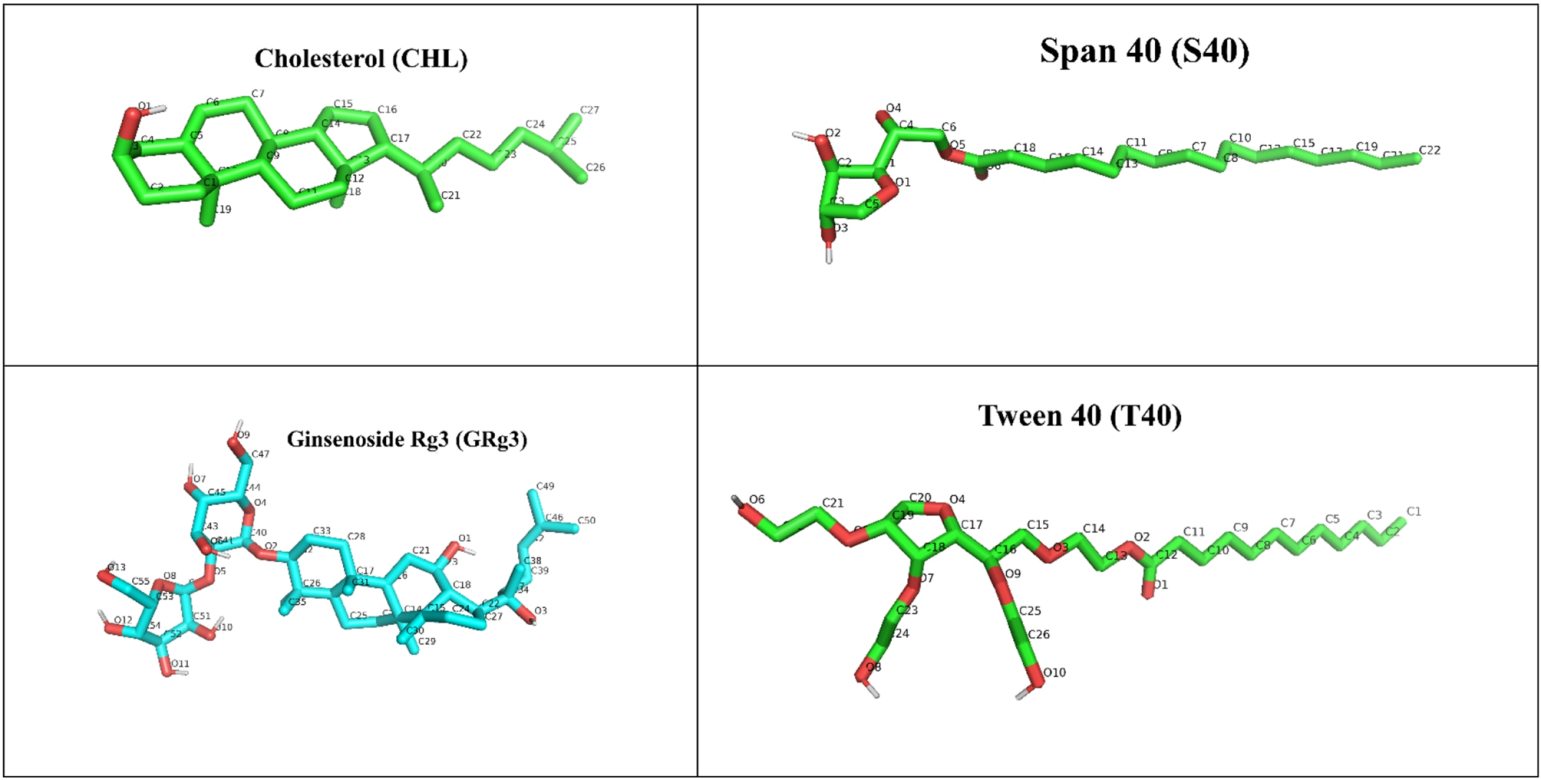
Atomic structures of T40, S40, CHL, and GRg3, annotated according to the Amber nomenclature.

MD simulations are essential tools for studying the self-assembly process of niosomes, offering detailed insights into the interactions at the atomic level. By utilizing MD simulations, we can observe how components such as T40, S40, CHL, and bioactive molecules like GRg3 interact and assemble spontaneously into stable bilayer structures. Figure 5 illustrates the atomic structures of T40, S40, CHL, and GRg3, annotated according to the Amber nomenclature. This detailed atomic labeling facilitates understanding the specific hydrogen bonding interactions that drive the stability and structural organization of the niosomal self-assembly process.

**Fig. 6:**
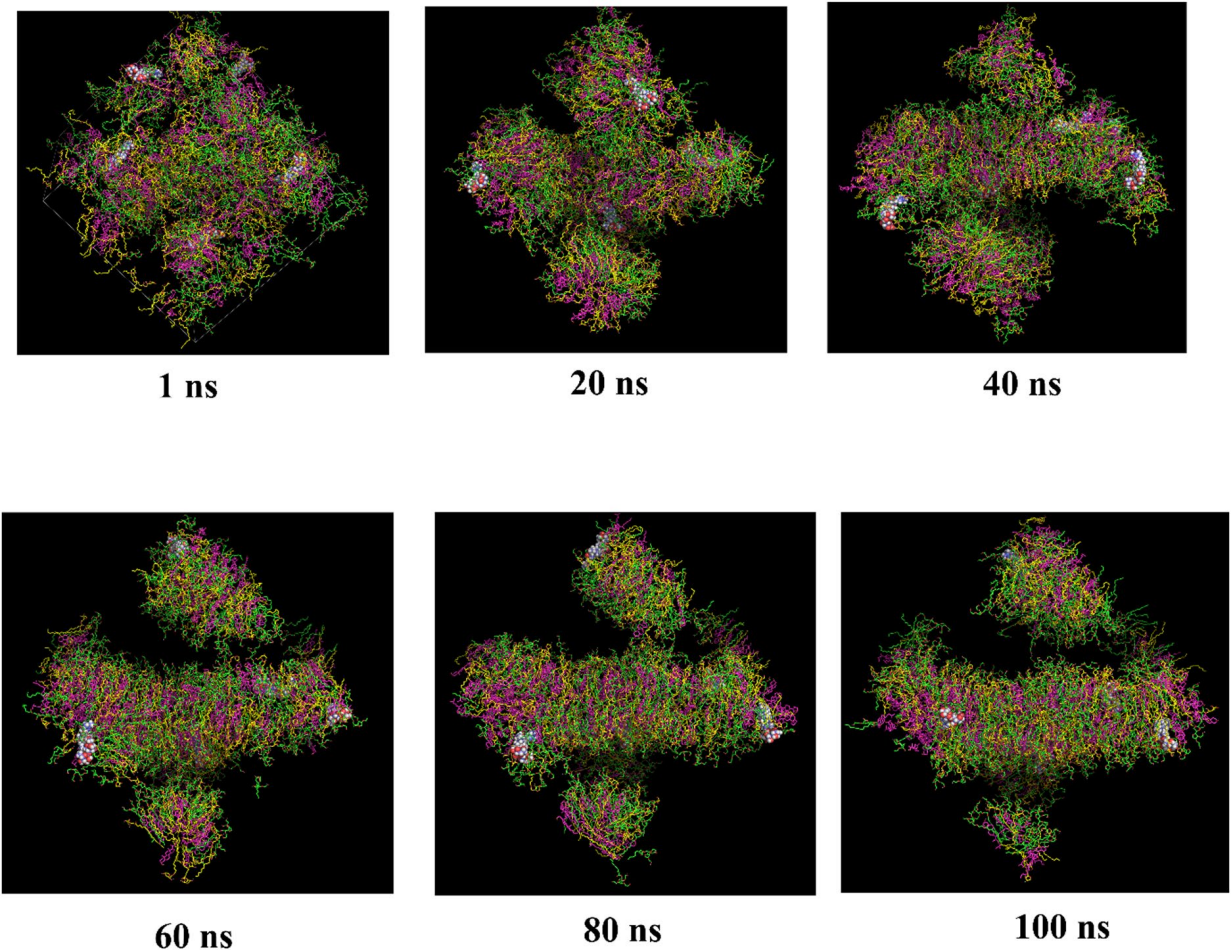
Snapshots of MD simulation depicting the time-dependent self-assembly of the niosomal bilayer system over 100 ns. The bilayer is composed of T40 (green), S40 (yellow), and CHL (magenta). The encapsulated GRg3 (sphere representation) is shown distributed within the forming bilayer, highlighting its stable integration at the hydrophobic core throughout the simulation timeframe.

Figure 6 demonstrates the self-assembly process of a niosomal bilayer composed of T40 (green), S40 (yellow), and CHL (magenta), encapsulating GRg3 (represented in sphere). Initially, at 20 ns, the amphiphilic components start clustering spontaneously due to hydrophobic interactions, progressively forming a defined bilayer structure by 60 ns. Throughout the simulation, the bilayer becomes increasingly organized, with T40 and S40 clearly oriented, displaying hydrophilic groups outward and hydrophobic tails inward, stabilized by CHL molecules. GRg3, the encapsulated drug, consistently localizes within the bilayer interface region rather than at the outer aqueous surfaces, signifying its stable entrapment within the hydrophobic core of the niosome. By 100 ns, the bilayer is fully assembled and stable, clearly encapsulating GRg3 within its central hydrophobic domain, which indicates effective self-assembly and potential for sustained delivery applications.

**Fig. 7:**
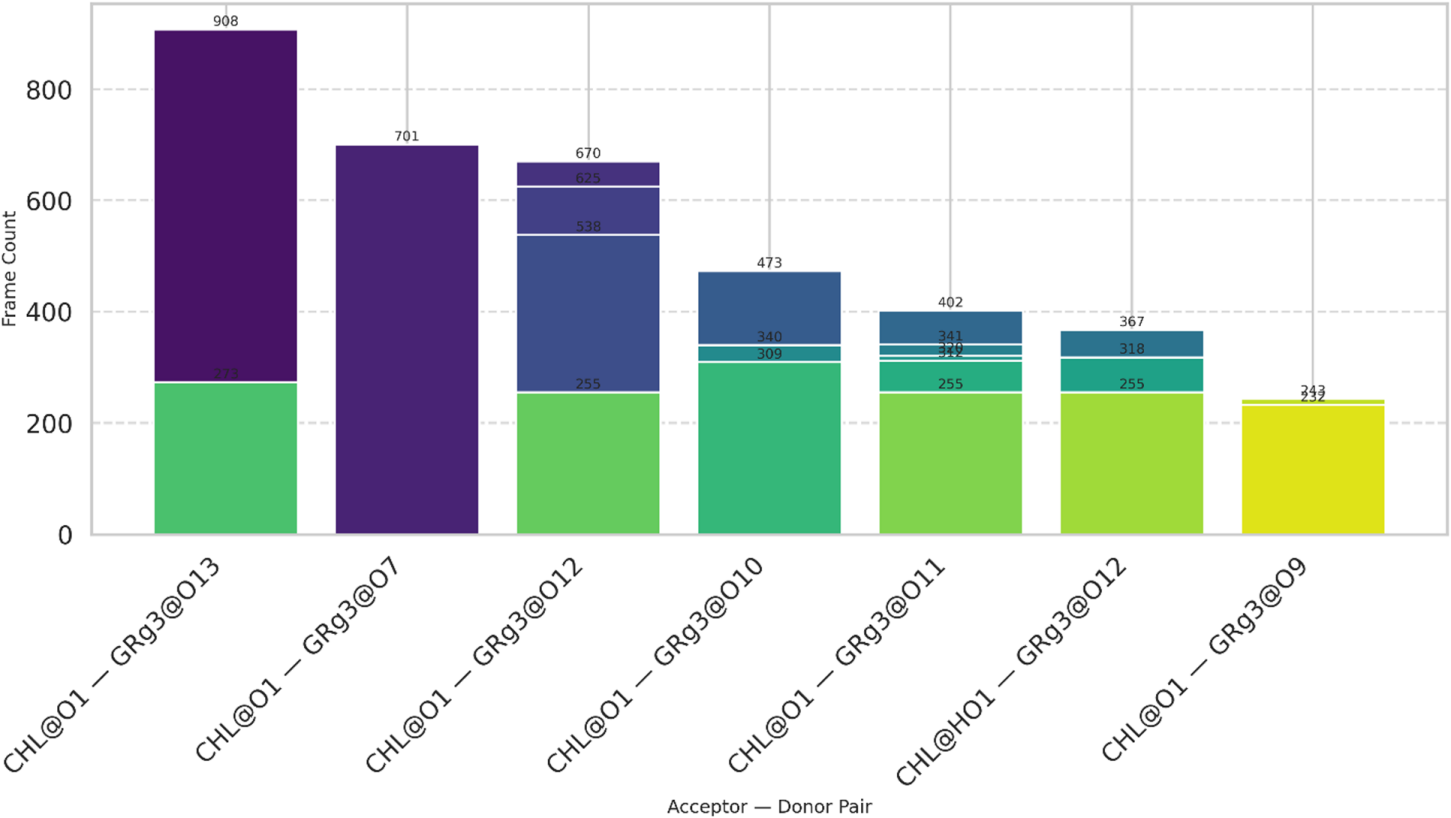
Hydrogen bond interactions observed between CHL and GRg3 during 100 ns MD simulations.

Hydrogen bonding significantly influences the stability and structural integrity of niosomal formulation by facilitating the interaction between the encapsulated drug and niosomal components. In the current analysis of GRg3 interaction with CHL, within the niosomal bilayer which has been shown in Fig. 7, several distinct hydrogen bond pairs were identified, predominantly involving the oxygen atoms of CHL (particularly O1 and occasionally HO1) acting as acceptors, and hydroxyl oxygens (O13, O7, O12, O10, O11, and O9) from GRg3 acting as hydrogen donors. According to Fig. 7, the most prominent interaction observed was between the O1 atom of CHL and the O13 atom of GRg3, present in 908 simulation frames, suggesting a remarkably stable and persistent interaction. Other notable hydrogen bonds include CHL@O1–GRg3@O7 and CHL@O1–GRg3@O12, reinforcing the critical role of hydroxyl groups on GRg3 in mediating strong intermolecular interactions. Such interactions underline the importance of specific atomic sites in drug encapsulation, potentially enhancing drug loading stability and bioavailability in therapeutic niosomes.

**Fig. 8:**
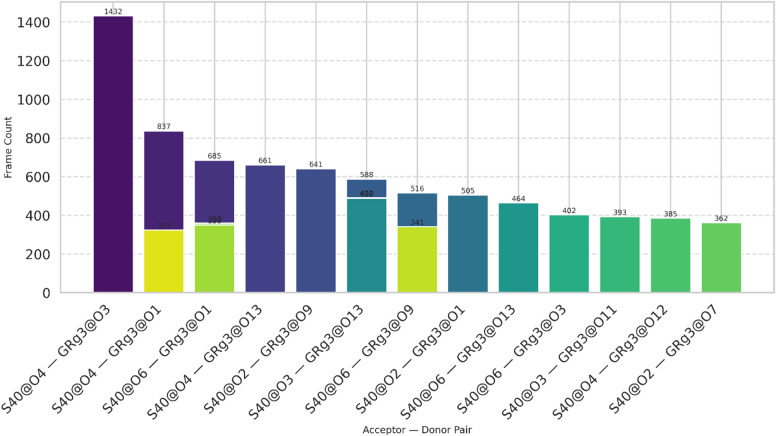
Hydrogen bonding analysis between S40 and GRg3, highlighting atom pairs like S40@O4–GRg3@O3 and S40@O4–GRg3@O1 as significant contributors.

The MD simulation analysis also revealed substantial hydrogen bonding between the GRg3 molecule and the S40 surfactant. The results have been shown in Fig. 8. The most frequent hydrogen bond observed was between the O3 atom of GRg3 and the O4 atom of S40, occurring in 1432 frames, indicating a strong and persistent interaction. Significant hydrogen bonding was also observed between GRg3@O1 and the S40@O4 atom, evident in 837 frames, suggesting that these sites also contribute notably to stability and interaction specificity. Other important hydrogen bond interactions include GRg3@O13 with S40@O4 and GRg3@O9 with S40@O2, further highlighting the role of specific oxygen atoms from both GRg3 and S40 in establishing robust intermolecular contacts. These interactions underscore the importance of specific functional groups within GRg3 and S40, which may influence the efficiency of drug encapsulation and release in niosomal formulations.

**Fig. 9:**
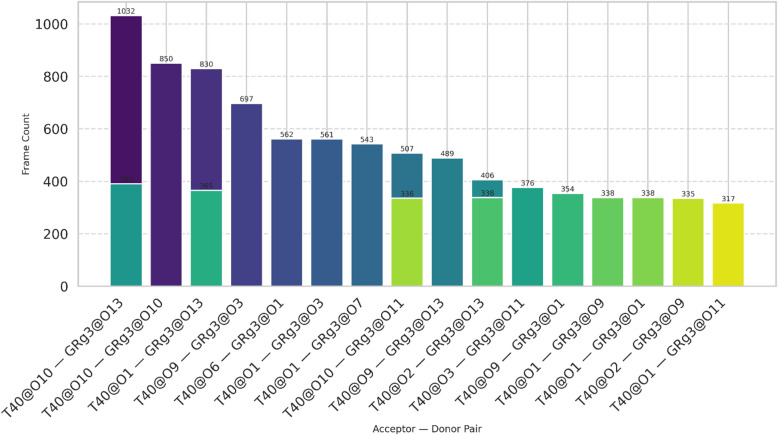
Dominant hydrogen bond interactions between T40 and GRg3 during MD simulations.

Similarly, as illustrated in Fig. 9, the GRg3 and T40 interaction profile indicates that the strongest hydrogen bonding interaction involves the pair T40@O10 and GRg3@O13, which appears in 1032 frames. Oxygen atoms O10, O1, and O9 from T40 and O13, O10, and O1 from GRg3 dominate these interactions. These strong, frequent hydrogen bonds substantially contribute to the stabilization and possibly affect the release properties of the encapsulated active compounds within the T40-based niosomal formulation.

## Discussion

In this study, the GRg3 metabolite was successfully incorporated into PEGylated niosomal carriers, and its neuroprotective efficacy was evaluated using both in vitro and in vivo models of AD. Complementary bioinformatics analyses were also employed to simulate and predict the molecular interactions between GRg3 and the niosomal phospholipid bilayer, as well as to estimate the efficiency of drug encapsulation and penetration within the nanocarrier system. The niosomes were formulated using the thin-film hydration technique, a widely utilized and effective method for preparing niosomal vesicles^43,44^. Based on DLS analysis, the observed increase in particle size for the PEGylated niosomes was attributed to the addition of a hydrated PEG layer on the vesicle surface, which extended the hydrodynamic diameter of the vesicles. The more negative zeta potential in the PEGylated formulation suggested that PEG incorporation also influenced surface charge, possibly due to the exposure of terminal hydroxyl or carboxyl groups, or changes in surface ion distribution. This negative charge enhanced electrostatic repulsion between vesicles, contributing to improved colloidal stability alongside the steric stabilization provided by the PEG chains.

The NPs sizes observed in this study were consistent with previous reports utilizing similar components, such as cholesterol, Span, or Tween, for niosome preparation. For instance, Agarwal et al. reported an optimal niosomal formulation containing cholesterol with a particle size of approximately 479 nm^45^. Likewise, research by Šturm et al. showed that the size of liposomal vesicles can vary widely, ranging from approximately 100 nm to over 500 nm, depending largely on the concentration of components such as cholesterol^46^. Similar findings were reported by Ghadi et al., who demonstrated that PEGylated niosomes exhibited increased particle size and a diffuse TEM boundary due to the PEG coating, thereby enhancing their in vivo stability and drug delivery performance^47^. Likewise, a study by Shahbazi et al. found that PEGylated niosomes exhibited larger diameters and improved stealth properties compared to their non-PEGylated counterparts, attributed to the extended PEG chains on their surface^48^. In this study, UV-vis spectroscopy was employed to assess the EE and in vitro release profile of GRg3, demonstrating that a substantial amount of GRg3 was successfully loaded into the niosomes. These results are consistent with those of Varshosaz et al., who reported an EE of 83% for ascorbic acid using niosomes formulated with Span and cholesterol^49^. Similarly, in a research involving PEG-PCL nanomicelles, the EE of GRg3 was found to be 80.6 ± 3.0%^50^. In another investigation by Wang et al., liposomes formulated with cholesterol, lecithin, and DSPE-PEG-GA for the co-delivery of GRg3 and ursolic acid achieved an EE of 71.68% for GRg3^51^. This sustained release pattern in the current investigation is consistent with findings from Yang et al., who reported approximately 80% release of GRg3 from liposomal formulations within the same timeframe^52^. In contrast, Wang et al., observed a complete release (100%) of GRg3 from PEGylated liposomes within 24 h^51^, indicating that different nanocarrier systems and their compositions can significantly influence drug release kinetics. The drug release kinetics observed in our study, characterized by a Korsmeyer–Peppas release exponent (n) of 0.447, indicate a Fickian diffusion mechanism. This finding aligns with several studies on niosomal drug delivery systems, where similar release behaviors have been reported. For instance, a study on aceclofenac-loaded niosomes reported n values ranging from 0.60 to 0.79, suggesting a release mechanism governed by both diffusion and erosion processes. Similarly, research on luteolin-loaded niosomes found n values between 0.25 and 0.71, indicating Fickian diffusion as the primary mechanism of release. These studies support the conclusion that niosomal formulations can effectively provide controlled drug release through diffusion-dominated mechanisms^53–55^. Furthermore, the high correlation coefficient (R² = 0.982) observed in our study for the Korsmeyer–Peppas model underscores the model’s suitability in describing the release kinetics of our niosomal formulation. This finding is consistent with those from other studies, where the Korsmeyer–Peppas model provided the best fit for drug release data from niosomal systems^56,57^. Several studies have investigated the development of nanoformulations containing ginseng-derived compounds for the treatment of AD and other disorders. For instance, Li et al. designed American ginseng-derived vesicle-like nanoplatforms to evaluate their anti-inflammatory effects in zebrafish and RAW264.7 macrophage cells. These nanoplatforms exhibited a mean particle size of 243 nm and a zeta potential of − 14.5 mV^58^. In another study, Zare-Zardini et al. developed a niosomal formulation of ginsenoside Rh2 (GRh2), which demonstrated a mean diameter of 93.5 nm, a zeta potential of + 4.65 mV, and an EE of 98%. These findings highlight the potential of niosomal systems as efficient carriers for delivering ginsenoside compounds^59^.

In addition to the physicochemical characterization of novel formulations, evaluating their biological efficacy at both the cellular and animal levels is crucial for determining their therapeutic potential. In the present study, we assessed the co-treatment efficacy of Aβ with niosomal formulations of GRg3. Our results indicated that an optimal concentration of 10 µmol/L was most effective for Aβ, free GRg3, and the PEGylated niosomal formulation. Notably, cells co-treated with niosomal GRg3 and Aβ exhibited higher viability compared to those treated with free GRg3 and Aβ. This enhanced viability may be attributed to the sustained drug release profile provided by the niosomal system. Consistent with our findings, previous studies have reported that nanocarrier-based drug delivery systems demonstrate superior efficacy compared to free drugs in in vitro cellular assays, such as the MTT assay. These improvements have been widely attributed to the controlled and sustained release behavior of nanocarrier formulations, which enhances cellular uptake and prolongs therapeutic activity^60,61^. It is noteworthy that the formulations in the present study did not exhibit an apparent concentration-dependent activity; however, the optimal concentration was identified within the tested range. A similar trend was reported by Chen et al., where treatment with free ginsenoside Rh (GRh) on M146L cells did not result in a concentration-dependent increase in cell viability. Instead, an optimal concentration was observed after 48 h of treatment, beyond which no further enhancement in viability was detected^62^.

Aβ is the primary protein involved in amyloid plaque formation in AD, with its neurotoxicity closely linked to the overproduction of ROS and lipid peroxidation^63^. Therefore, measuring lipid peroxidation and MDA levels presents a promising approach for identifying the efficacy of AD treatments^36^. In the current study, lipid peroxidation, measured by MDA levels, was significantly reduced in the Aβ + PEGylated GRg3-loaded niosome-treated group compared to the Aβ-treated group.

The antioxidant activity of P. ginseng and its derivatives has also been confirmed in other conditions, including heart failure, rheumatoid arthritis, and dexamethasone-induced oxidative stress. In a study conducted by Du et al., ginsenoside Rb1-loaded PLGA NPs exerted a significant effect on MDA levels in the H9c2 cell line, indicating their potential antioxidant activity^64^. The therapeutic potential of ginsenosides has been demonstrated not only in cellular assays but also in serum analyses from animal models. For instance, Liu et al. reported that both free ginsenoside Rb1 and its NPs formulation effectively reduced serum MDA levels in a rheumatoid arthritis model, with the nanoplatform showing superior efficacy compared to the free drug^65^. Similarly, Abdel Aziz et al. found that treatment with nano-ginseng significantly decreased serum MDA levels and increased TAC in rats, outperforming both the control and dexamethasone-treated groups^66^.

However, in the current study, TAC remained relatively unchanged among the treatment groups, suggesting that although GRg3 exhibits antioxidant properties, it may not be sufficient to fully restore the oxidative balance disrupted by Aβ in neuroblastoma cells under the tested conditions. Both the free and PEGylated GRg3-loaded niosome forms were unable to completely reverse Aβ-induced oxidative damage, highlighting certain limitations in their antioxidant therapeutic potential^67–69^. Consistent with this observation, Singh et al. reported that GRg3 (at concentrations ranging from 1 to 100 µg/mL) had no significant effect on reducing ROS generation in RAW 264.7 murine macrophage cells. However, in their investigation, when GRg3 was conjugated with a nanocarrier, specifically superparamagnetic iron oxide nanoparticles (SPIONs), a marked reduction in ROS levels was observed^70^. Furthermore, in the mentioned study, ginsenoside CK (a type of ginseng derivative) demonstrated higher antioxidant activity than GRg3, indicating variations in the antioxidant activity of its substance^70^.

*Caspase-3*, a pivotal enzyme within the caspase family, plays a central role in the execution of programmed cell death (apoptosis). In AD patients, *caspase-3* immunoreactivity is markedly increased in neurons and astrocytes, as well as within neurofibrillary tangles and amyloid plaques^86^. The accumulation of Aβ has been shown to activate *caspase-3*, thereby promoting neuronal apoptosis and facilitating the formation of neurofibrillary tangles. These processes collectively contribute to neuronal degeneration and the progression of Aβ plaque pathology in the AD brain^20^. Nonetheless, analysis of *caspase-3* expression revealed that the PEGylated GRg3-loaded niosomal NPs more effectively suppressed early apoptosis triggered by Aβ compared to the free form, indicating that GRg3, particularly in its niosomal form, can attenuate Aβ-induced apoptotic signaling. In a study by Li et al., the efficacy of ginsenoside Ro (Gro) in modulating *caspase-3* gene expression was demonstrated in APP/PS1 transgenic mice, an established AD model, as evidenced by Western blot analysis. This modulation is closely associated with the regulation of apoptosis in neuronal cells^18^. Consistent with these findings, our results confirm that both free and niosomal formulations of GRg3 induce apoptosis in neuronal cells, a therapeutic attribute that may contribute positively to the treatment of AD^71^.

Although the antioxidant activity of the formulation was partially preserved, the results of the MDA assay, MTT assay, and *caspase-3* expression analysis collectively indicated that the designed niosomal formulation exerted a beneficial effect on neuronal cells in vitro. Following these evaluations, the efficacy of the formulations was further examined using an AD model. The enhanced anxiolytic effect and behavioral normalization seen with the PEGylated formulation align with prior evidence of improved outcomes from CNS-targeted nano-delivery systems. Despite these promising findings, limitations must be acknowledged. Behavioral tests such as the OFT may conflate motor and affective components, making it challenging to isolate anxiety-related behaviors. To address this, the use of complementary tests, such as the MWM and Y-maze, enhances the robustness of cognitive assessments and minimizes confounding factors related to locomotor activity. Future studies should incorporate additional behavioral paradigms and long-term assessments to evaluate sustained efficacy and safety. Behavioral assessments revealed that Aβ exposure significantly impaired spatial working memory and exploratory behavior, consistent with known models of AD-like pathology. Notably, both GRg3 formulations reversed these deficits as evidenced by increased spontaneous alternation in the Y-maze test and enhanced performance in the MWM, suggesting improved hippocampal function and synaptic plasticity. The restoration of rearing and grooming behaviors following free GRg3 treatment may indicate modulation of dopaminergic signaling. At the same time, PEGylated GRg3 exhibited superior anxiolytic effects, as reflected by increased center exploration in the OFT. These observations underscore GRg3’s neuromodulatory potential and the role of PEGylation in enhancing bioavailability and CNS penetration^72^. Other studies also indicated the professional effect of ginsenosides on the activity and behavioral approach of AD models. Zhang et al. demonstrated that the moving distance and speed were reduced meaningfully in AD mice receiving ginsenoside RG1 compared to the AD model^73^. In an investigation by Nie et al., mice treated with GRg1 exhibited time spent and distance traveled in the center of the open-field assay comparable to those of wild-type controls^84^, findings that are consistent with the results of the present study. Another investigation demonstrated a progressive decrease in escape latency from day 1 to day 4 in mice treated with ginsenoside, indicating improved learning and memory performance. Interestingly, the swimming time spent in the target quadrant by the treated group was comparable to that of wild-type mice, suggesting enhanced spatial memory retention^73^. Similarly, Nie et al. reported that mice administered GRg1 exhibited escape latency times closely resembling those of wild-type controls. Moreover, the number of platform crossings and probe trial duration in the Morris Water Maze further confirmed memory improvement in the treatment group^74^.

In addition to behavioral analysis, evaluating brain histological changes and measuring oxidative stress levels in brain tissue can provide deeper insights into the therapeutic potential and efficacy of the formulation. Histological assessments can reveal the extent of neuronal protection, inflammation, and tissue integrity, while oxidative stress markers help determine the formulation’s ability to counteract neurodegenerative damage at the molecular level^61^. Together, these evaluations contribute to a more comprehensive understanding of the formulation’s impact on AD pathology. Aβ administration disrupted BBB integrity, a hallmark of early neurodegeneration, whereas treatment with either GRg3 formulation preserved normal neurovascular architecture. This neurovascular protection likely stems from the compound’s antioxidative and anti-inflammatory properties. In the present study, mechanistically, PEGylated GRg3 reduced hippocampal MDA levels, suggesting a potent antioxidant capacity potentially mediated by ROS scavenging or activation of the Nrf2 pathway. In addition, decreased expression of glial fibrillary acidic protein (GFAP) and ionized calcium-binding adapter molecule 1 (Iba1) following treatment indicates suppression of astrocytic and microglial activation, respectively—hallmarks of neuroinflammation. These effects are consistent with NF-κB pathway inhibition, further supporting GRg3’s immunomodulatory role^75,76^. This immunohistochemical analysis demonstrates that the niosomal formulation significantly mitigates Aβ-induced hippocampal pathology compared to free GRg3, as evidenced by distinct histopathological differences between the groups. While Aβ infusion alone induced characteristic AD-like damage, including substantial plaque deposition and disrupted cytoarchitecture, treatment with PEGylated GRg3-niosomes promoted visible improvements in tissue morphology, such as nuclear enlargement and reduced dendritic atrophy, suggesting partial preservation of neuronal integrity and health. Crucially, these benefits were not observed with free GRg3 administration, which was associated with persistent signs of neuronal stress, notably increased perineuronal space. These findings strongly indicate that the pegylated niosomal formulation enhances the therapeutic efficacy of GRg3, likely by improving its bioavailability, stability, and/or targeted delivery to the affected hippocampal tissue, thereby offering superior neuroprotection against Aβ toxicity. It is safe to assume that PEGylation enhanced the pharmacokinetic profile of GRg3 by reducing immune clearance and prolonging systemic circulation, resulting in sustained therapeutic exposure. These characteristics are critical for chronic neurodegenerative conditions, where consistent drug delivery across the BBB remains a key challenge^77^.

In addition, we would like to emphasize that the composition of our PEGylated niosomes was specifically optimized to favor brain delivery. Previous reports have demonstrated that PEGylation enhances systemic stability and prolongs circulation time, increasing the likelihood of interaction with the BBB endothelium and facilitating transport through adsorptive or receptor-mediated mechanisms^78,79^. Moreover, in this study, we employed PEG with a molecular weight of 6 kDa for surface modification of the niosomal vesicles. The PEG chain length plays a crucial role in determining nanoparticle circulation, biodistribution, and BBB permeability. Previous studies have demonstrated that long-chain PEGs (≥ 5 kDa) provide prolonged systemic circulation, thereby increasing the probability of nanoparticle interaction with the BBB endothelium. Interestingly, in vivo investigations have shown that liposomes modified with long-chain PEGs exhibit enhanced brain accumulation in both healthy and GBM models, consistent with their in vitro BBB penetration profiles. This improvement was attributed to the combined effects of prolonged systemic retention and increased BBB transport efficiency associated with long-chain PEGylation^80,81^. Therefore, the use of PEG 6 kDa in our formulation was intended to maximize systemic persistence and facilitate potential brain delivery through these synergistic mechanisms. Moreover, the cholesterol component contributes to membrane rigidity and stability, whereas the Span 40/Tween 40 surfactant combination modulates vesicle fluidity and surface hydrophilicity, both of which are known to influence BBB permeability^82,83^. Moreover, Tween surfactants (mainly Tween 80) are widely recognized as one of the “gold-standard” excipients for enhancing BBB delivery. They promote nanoparticle transport across the BBB through multiple mechanisms, including adsorptive-mediated and apolipoprotein (ApoE and ApoB)-mediated endocytosis, which mimic low-density lipoprotein (LDL) receptor pathways^26,61,84,85^.

Among them, Tween 40 has been shown to improve brain uptake of NPs not only by facilitating surface adsorption of plasma apolipoproteins but also by inhibiting P-glycoprotein (P-gp) efflux activity, thereby reducing active drug extrusion from endothelial cells^82,86^. These findings support the hypothesis that the mixed composition of Tween 40, Span 40, and PEG in our niosomal composition could contribute to enhanced BBB permeation despite the relatively large particle size. Therefore, although direct BBB transport quantification was beyond the scope of this initial study, our formulation design incorporated structural features previously correlated with enhanced brain accumulation of nanosystems. Furthermore, the ability of NPs to cross the blood–brain barrier (BBB) is strongly influenced by their physicochemical properties, particularly particle size, surface chemistry, and structural flexibility^87,88^. Previous studies have shown that nanosystems with diameters below approximately 200 nm are generally more favorable for transcytosis or paracellular diffusion across the BBB, as smaller sizes facilitate endothelial uptake and reduce recognition by the reticuloendothelial system^77,89,90^. We acknowledge that in our study, the PEGylated niosomes exhibited hydrodynamic diameters with peaks around 800–900 nm, reflecting an increase in apparent size due to PEG surface modification and hydration. Although this value exceeds the typical range for optimal BBB transfer, the soft, deformable lipid-based structure of niosomes may allow transient shape adaptation under shear stress or during endothelial interaction, facilitating partial BBB penetration despite the larger measured size^87,91,92^. Furthermore, in our study, immunohistochemical analysis revealed a marked reduction in Aβ deposition in the brain tissues of treated animals, indicating that the niosomal formulation exerted a therapeutic effect within the central nervous system. This outcome indirectly supports the hypothesis that the formulation, or its released drug, reached the brain parenchyma and was biologically active against Alzheimer’s pathology. However, we acknowledge that direct quantitative and qualitative investigations to track vesicular distribution across the BBB—such as fluorescence imaging, radiolabeling, or confocal microscopy—were not performed in this proof-of-concept study. This limitation should be acknowledged, and future studies should focus on conducting in vivo biodistribution and vesicle tracking experiments to verify the extent of BBB penetration and brain localization.

The neuroprotective effects of ginseng compounds, particularly GRg2 and GRh1, have been comprehensively reviewed by Liu et al. They reported that, beyond their pharmacological activities in AD treatment and mitigation of memory loss, these compounds also exert histopathological benefits. These include the protection of nerve cells, alleviation of neuronal injury and ischemia, maintenance of vascular integrity, and reduction of neuronal apoptosis, mediated through multiple signaling pathways, such as the PI3K/Akt pathway^93^. In another study, Chu et al. demonstrated that administration of GRg5 (5–20 mg/kg) to AD model rats resulted in a dose-dependent reduction of Aβ concentrations in the hippocampus and cerebral cortex^94^. Similarly, Shin et al. showed that treatment with red ginseng extract in AD mice preserved neuronal cell integrity, mitigated mitochondrial activation in neurons, and reduced Aβ accumulation in the subiculum. Immunofluorescence analysis further revealed a significant decrease in GFAP-positive areas, indicating reduced astrocyte activation in the treatment group^95^. Furthermore, Li et al. reported a decline in both GFAP-positive areas and Aβ levels following ginseng compound treatment, reinforcing the anti-inflammatory and neuroprotective properties of ginseng derivatives in AD models^18^. Ramesh et al. demonstrated that oxidative stress was alleviated by an increase in SOD levels in the hearts, livers, kidneys, and lungs of aged rats^96^. Therefore, enhancement of SOD activity in organs is considered a promising indicator of antioxidant efficacy. Further studies have reported the beneficial effects of nano-ginseng and ginsenoside Rb1 in reducing MDA levels in serum^65,66^. Additionally, a study by Kim et al. found that CAT content in the blood was significantly elevated in mice treated with Wild Ginseng Root Extract compared to the control group^97^. Zhang et al., further demonstrated that the activities of MDA, SOD, and CAT vary depending on the specific ginseng compounds; for example, Rg1 increases SOD activity and reduces MDA levels, whereas Rg3 enhances CAT and SOD activities while decreasing MDA content^98^., which is in accordance with the results of our study. These findings provide insight into the multifaceted mechanisms by which GRg3 confers neuroprotection, highlighting the advantages of PEGylated nanoformulations for CNS drug delivery^99^.

The data presented in the current study align with previous research on ginseng-based therapies for AD, underscoring the therapeutic potential of the developed formulation. Notably, our findings demonstrated that the niosomal formulation exhibited enhanced efficacy, highlighting the advantage of nanocarriers in the targeted delivery of encapsulated compounds. Future research should also explore the co-delivery or combination of ginseng derivatives with existing standard treatments for AD, as such synergistic approaches may enhance therapeutic efficacy and overcome limitations of monotherapies^86^.

MD simulations provide critical insights into the self-assembly behavior of niosomal systems composed of Tween 40 (T40), Span 40 (S40), cholesterol (CHL), and the bioactive compound ginsenoside GRG3. Over the course of a 100 ns simulation, the spontaneous formation of a stable bilayer was observed, marked by progressive clustering and orientation of amphiphilic components. Analysis of hydrogen bonding revealed significant interactions contributing to this stability, with cholesterol predominantly engaging GRG3 via its O1 atom, particularly forming stable bonds with GRG3@O13 and GRG3@O7 atoms. Additionally, Span 40 displayed strong hydrogen bonding primarily through its O4 and O6 atoms, frequently interacting with GRG3@O3 and GRG3@O1, suggesting effective incorporation and stabilization of GRG3 within the Span 40-rich regions of the bilayer. Tween 40 similarly exhibited prominent hydrogen bonding interactions with GRG3 via its O10 atom, notably interacting with GRG3@O13 and GRG3@O10, thereby reinforcing GRG3’s encapsulation within the bilayer. Throughout the simulation, GRG3 molecules predominantly positioned themselves at the bilayer interface, interacting robustly with surrounding amphiphiles. These detailed interactions illustrate the molecular-level mechanisms by which GRG3 is stably incorporated into the niosome structure, highlighting the critical role of specific hydrogen bonds in maintaining structural integrity and drug encapsulation efficiency. Further studies have employed MD simulations to elucidate the interactions between ginseng compounds and key biomolecules, demonstrating interactions with interleukin and *caspase-3*^100^. In this context, the formation of nanocarriers such as niosomes has been explored in silico. For instance, Khaleghian et al. conducted MD investigations of curcumin-loaded niosomes and confirmed that hydrophobic interactions play a critical role in curcumin encapsulation^101^. In the present study, for the first time, the ginseng compound GRg3 was encapsulated within PEGylated niosomes. The encapsulation process and the formation of this novel niosomal system were characterized using MD simulations, revealing an efficient mechanism for GRg3 delivery.

## Conclusion

This study underscores the promising potential of PEGylated niosomal formulations as effective delivery systems for GRg3 in the treatment of AD. By achieving high EE and controlled release, this nanocarrier enhances the bioavailability and therapeutic efficacy of GRg3. The observed neuroprotective and anxiolytic effects, likely facilitated by improved BBB penetration and antioxidative mechanisms, highlight the formulation’s ability to target key pathological features of AD. Additionally, MD simulations provide valuable mechanistic insight into the stable self-assembly and drug–carrier interactions at the molecular level, informing the rational design of future niosomal systems. Collectively, these findings contribute to the growing body of evidence supporting nanotechnology-based approaches to improve phytochemical delivery and offer new avenues for AD intervention.

Future research should focus on in-depth pharmacokinetic and pharmacodynamic studies in relevant animal models to further elucidate the in vivo behavior and long-term safety of PEGylated GRg3-loaded niosomes, as well as in vivo biodistribution and vesicle tracking analyses to confirm the extent of BBB penetration and brain localization. Exploring the potential for targeted delivery through surface modification with ligands or antibodies may enhance specificity and therapeutic outcomes. Moreover, clinical translation will require evaluation of the scalability, stability, and regulatory compliance of the formulation. Ultimately, integrating such nanocarriers with multi-modal therapeutic strategies could pave the way for more effective and personalized treatments for Alzheimer’s disease.

## Supplementary Information

Below is the link to the electronic supplementary material.

Supplementary Material 1

## Data Availability

The datasets generated and/or analyzed during the current study are not publicly available but are available from the corresponding author on reasonable request.
