# Supplementary figures and images for: Ginsenoside Rg3-encapsulated pegylated niosomes exhibit multimodal therapeutic potential in Alzheimer’s disease
Source: Sci Rep. 2025 Dec 14;16:2547. doi: 10.1038/s41598-025-32528-3 (PMC12820278; doi:10.1038/s41598-025-32528-3)

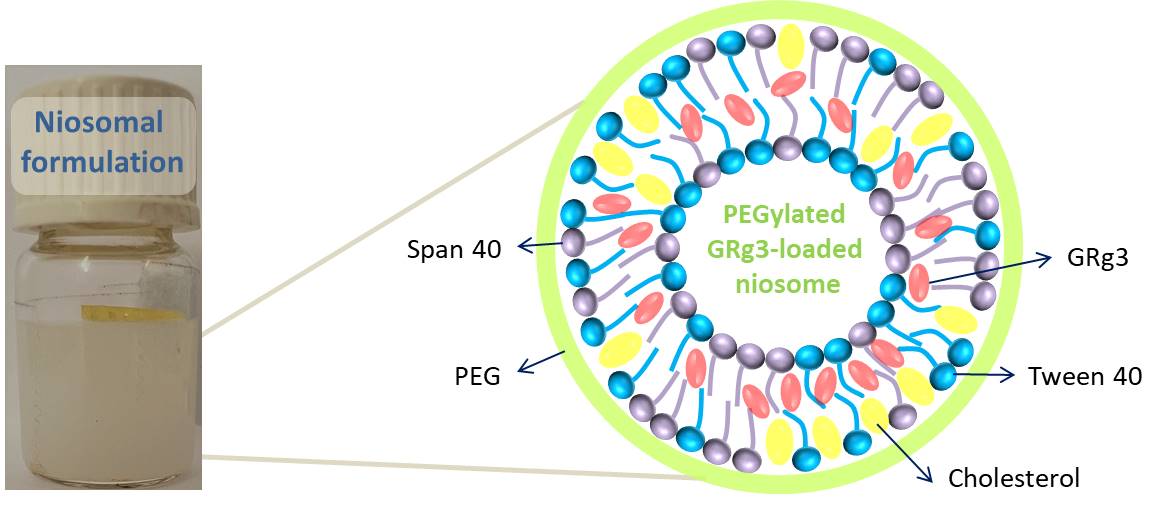


**Figure S1.** Prepared niosomal formulation.

Supplement: Supplementary file 1 — Supplementary Material 1 [file 41598_2025_32528_MOESM1_ESM.docx]
